# SOCS2 Binds to and Regulates EphA2 through Multiple Mechanisms

**DOI:** 10.1038/s41598-017-11040-3

**Published:** 2017-09-07

**Authors:** Carissa Pilling, Jonathan A. Cooper

**Affiliations:** 1 Division of Basic Sciences, Fred Hutchinson Cancer Research Center, 1100 Fairview Ave N, Seattle, Washington, 98109 USA; 2 Molecular and Cellular Biology Program, 1959 NE Pacific Street, HSB T-466, University of Washington, Box 357275, Seattle, WA 98195-7275 USA

## Abstract

Suppressors of cytokine signaling (SOCS) proteins inhibit signaling by serving as substrate receptors for the Cullin5-RING E3 ubiquitin ligase (CRL5) and through a variety of CRL5-independent mechanisms. CRL5, SOCS2 and SOCS6 are implicated in suppressing transformation of epithelial cells. We identified cell proteins that interact with SOCS2 and SOCS6 using two parallel proteomics techniques: BioID and Flag affinity purification mass spectrometry. The receptor tyrosine kinase ephrin type-A receptor 2 (EphA2) was identified as a SOCS2-interacting protein. SOCS2-EphA2 binding requires the SOCS2 SH2 domain and EphA2 activation loop autophosphorylation, which is stimulated by Ephrin A1 (EfnA1) or by phosphotyrosine phosphatase inhibition. Surprisingly, EfnA1-stimulated EphA2-SOCS2 binding is delayed until EphA2 has been internalized into endosomes. This suggests that SOCS2 binds to EphA2 in the context of endosomal membranes. We also found that SOCS2 overexpression decreases steady state levels of EphA2, consistent with increased EphA2 degradation. This effect is indirect: SOCS2 induces EfnA1 expression, and EfnA1 induces EphA2 down-regulation. Other RTKs have been reported to bind, and be regulated by, over-expressed SOCS proteins. Our data suggest that SOCS protein over-expression may regulate receptor tyrosine kinases through indirect and direct mechanisms.

## Introduction

Suppressor of Cytokine Signaling (SOCS) proteins, SOCS1-7 and CisH, are classic negative regulators of cytokine signaling. SOCS proteins have an unstructured N-terminus of unknown function, a central SH2 domain that binds tyrosine-phosphorylated substrates, and a C-terminal SOCS-BC box. The latter mediates binding to the E3 ubiquitin ligase Cullin5 (Cul5) and adaptor proteins Elongin B and C (ElgB/C) to form a Cullin5-RING ligase (CRL5) complex^1^. Several SOCS genes are induced after acute cytokine stimulation, and the SOCS proteins inhibit JAK/STAT signaling through a variety of CRL5-dependent and independent mechanisms, including direct kinase inhibition and competition with other signaling proteins for binding sites on receptors^2, 3^.

SOCS proteins have also been reported to regulate JAK/STAT-independent signaling^1, 4, 5^. For example, SOCS proteins have been shown to bind to and negatively regulate receptor tyrosine kinases (RTKs) and their downstream signaling in multiple cell types^5^. Overexpression of SOCS proteins can slow RTK-dependent growth in the presence of RTK ligands^6–9^, and decrease ligand-induced activation of downstream signaling pathways^10–14^. For some RTKs, SOCS overexpression increases RTK ubiquitylation and decreases RTK expression^7, 12, 15–18^. One idea is that SOCS proteins recruit CRL5 to active RTKs in order to down-regulate RTK signaling. Classically, RTK signaling is down-regulated by the ubiquitylation of the RTKs and associated proteins, leading to internalization into early endosomes^19, 20^. Early endosomes then mature and undergo acidification, which may cause the associated ligand to be released from the receptor^19^. If ligand dissociates, the empty receptor may be de-ubiquitylated and recycled back to the plasma membrane. However, if the ligand remains bound, the RTK may remain ubiquitylated and may be captured by the ubiquitin binding domains of the endosomal-sorting-complex-required-for-transport (ESCRT) complex, and bud off into intra-luminal vesicles of the multi-vesicular bodies (MVBs)^19–21^. MVBs eventually fuse with the lysosome resulting in degradation of the RTK^22^. Continued ubiquitylation is critical for this process and CRL5^SOCS^ complexes could be involved.

The Eph receptors are the largest family of receptor tyrosine kinases (RTKs)^23^. They can be classified into EphA and EphB groups, with EphA’s preferentially binding to GPI-linked EphrinA (EfnA) ligands and EphB’s binding to transmembrane EfnB ligands^24^. Efn binding induces the formation of dimers and higher order clusters of active Eph’s that regulate cell responses, including cell motility, formation of tissue/cell boundaries, and proliferation^25^. After ligand binding, Eph receptors are trans-autophosphorylated at two conserved tyrosines in the juxtamembrane segment of the receptor. This is then followed by autophosphorylation of a tyrosine residue in the kinase activation loop, causing full activation of the receptor^26^.

We previously reported that inhibiting expression of Cul5 or a combination of SOCS 2, 4, 5, and 6 proteins induces growth factor-independent proliferation and migration of mammary epithelial cells in culture^27^. While we have identified one CRL5^SOCS6^ target, p130Cas, that is required for this phenotype, other targets remain to be discovered^27–29^. The question remains as to which tyrosine-phosphorylated proteins are regulated by CRL5 and mediate the altered biology of Cul5-depleted cells. Here we performed proteomics screens for cell proteins that interact with SOCS2 and 6, followed by an in-depth study of the interaction of EphA2 with SOCS2 that is stimulated by the EphA2 ligand, EfnA1. The results show that SOCS2 regulates EphA2 by direct and indirect mechanisms.

## Results

### Identification of SOCS2- and SOCS6-interacting proteins

To identify SOCS2 and 6-interacting proteins, we used two affinity purification mass spectrometry (AP-MS) methods: BioID and Flag AP-MS^30, 31^. For BioID, a bait protein is fused to a promiscuous, mutated, biotin ligase, BirA^30^. Biotin is then transferred to neighboring proteins, which may be isolated using streptavidin and identified using LC-MS/MS. Flag AP-MS is a more conventional affinity purification procedure in which the bait is fused to a Triple-Flag epitope tag and bound proteins are purified over anti-Flag antibody. These two methods identify overlapping sets of targets and have been used jointly to identify substrates for another CRL, CRL1^βTrCP^ ^31–33^.

Initial attempts to stably express tagged forms of SOCS2 and SOCS6 in HEK293 cells were unsuccessful due to strong selection against stable expression. We therefore made use of Flp-In T-Rex HEK293 cells, in which protein expression may be induced with doxycyline. We generated cell lines expressing SOCS2 and SOCS6 bearing Myc-BirA and Triple Flag tags, as well as Myc-BirA and Triple Flag tag vector cell lines as negative controls. Three biological replicates of SOCS2, SOCS6 and vector alone were performed for each procedure. The cells were incubated with doxycycline to induce expression of the bait proteins, and sodium orthovandate was added to globally increase tyrosine phosphorylation. In addition, we added MLN4924 to inhibit CRL activity and thereby stabilize CRL substrates from ubiquitylation and degradation^34^. The cells were lysed and the biotinylated or Flag proteins were purified. The proteins were eluted with on-bead trypsin digestion and analyzed with liquid chromatography-tandem mass spectrometry (LC-MS/MS). Peptides were identified and quantified using label-free quantification in MaxQuant^35^. We estimated the abundance of each protein in each replicate by averaging the quantities of its top three peptides^36^. The protein abundances from biological triplicate experiments were then analyzed statistically using SAINTq software^37^. For each protein detected, abundances from the bait of interest were compared with those from the other bait and negative control, and P value, Bayesian False Discovery Rate (FDR), and average fold change (FC) were reported (see Supplementary Dataset File). Here we consider the interactions that had a Bayesian False Discovery rate of less than ten percent as being significant.

**Figure 1:**
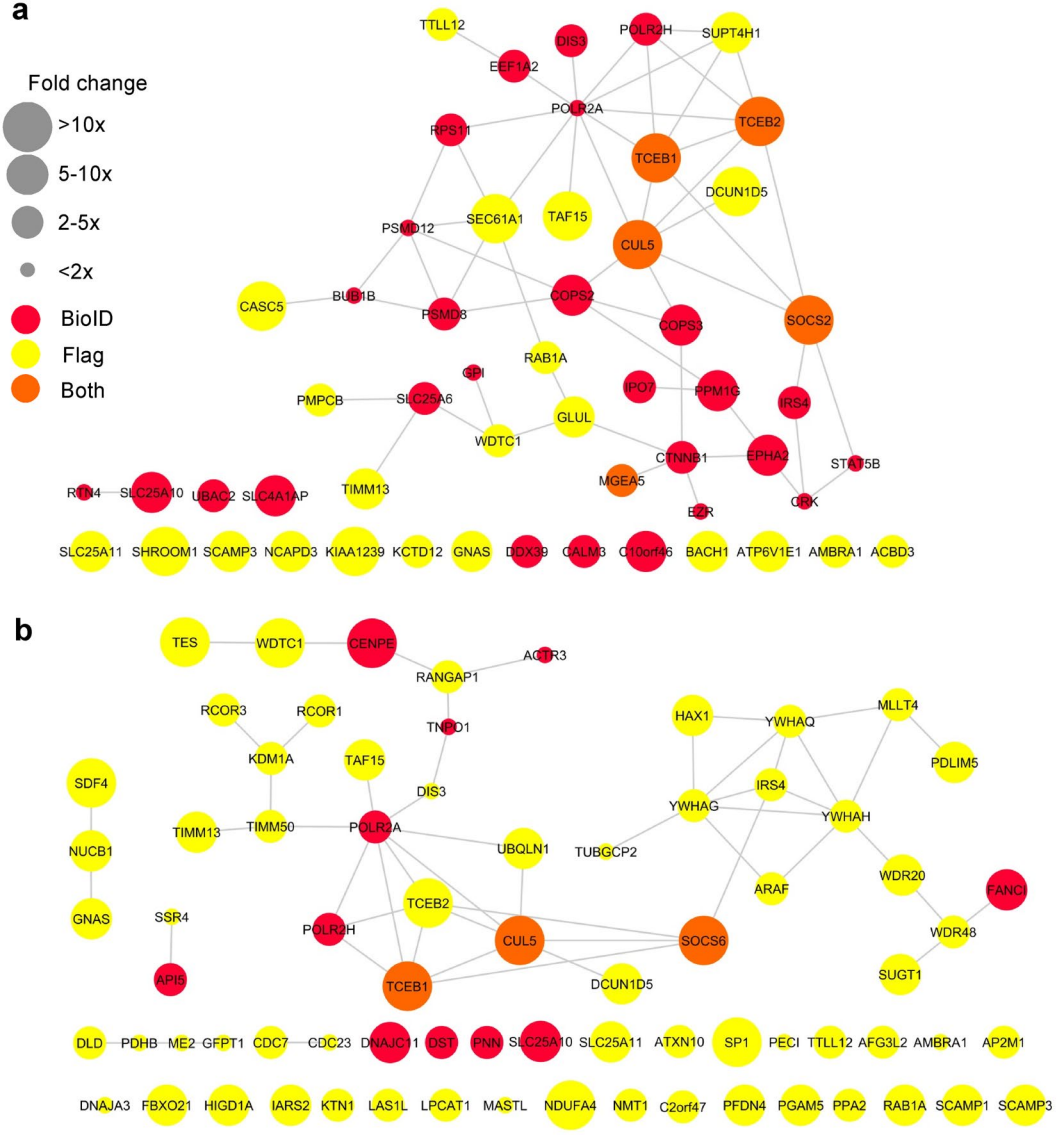
BioID and affinity purification MS identifies SOCS2 and SOCS6 interactomes. (**a** and **b**) SOCS2 and SOCS6 interactions identified using BioID and Triple-FLAG affinity purification mass spectrometry, with previously identified interactions added from the STRING database. Triple-FLAG or Myc-BirA tagged empty vector, SOCS2, or SOCS6 were stably integrated into Flp-In T-Rex 293 cells. The cells were treated with 75 µM sodium orthovandate, 1 µM MLN4924, 1 µg/mL Doxycycline and 2 mM Biotin for 24 hr, lysed and immunoprecipitated with either Flag M2 magnetic beads (Flag) or streptavidin agarose beads (BirA). An on-bead tryptic digestion was performed, followed by sample desalting. The samples were run on a Orbitrap Elite and peptides were identified using MaxQuant label-free quantification. The average of the top three peptide intensities from each biological replicate were used to estimate protein abundance and then run through SAINTq program. Interacting proteins shown had a Bayesian false discovery rate <10%. The STRING app in Cytoscape was used to determine the interactions between the top hits and make the figure. Full data are provided in the Supplementary Dataset File.

Figure 1 shows that 53 proteins interacted significantly with SOCS2 and 73 proteins interacted with SOCS6, colored according to their detection by BioID (red), Flag AP-MS (yellow) or both procedures (orange). The size of the symbol indicates the average FC over negative control from the biological triplicates. The lines represent protein-protein interactions previously reported in the STRING database^38^. The two proteomics methods are complementary, identifying different sets of interacting proteins. The Flag-AP-MS method identifies strong, easily solubilized interactions while the BioID method captures weaker, poorly solubilized proteins^31, 32^. However, some proteins were identified by both methods, including the CRL5 complex members Cul5, TCEB1 (ElgC) and TCEB2 (ElgB), which were doubly identified with SOCS2 and SOCS6. This shows that both methods were able to identify known components of the CRL5 complex.

Other proteins implicated in general ubiquitylation or proteasome functions were detected as SOCS2 or 6 interactors. Both SOCS2 and 6 interacted with DCUN1D5 (DCNL5), a member of the DCN family of E3 Nedd8 ligases that transfer Nedd8 onto the Cullin backbone^39, 40^. SOCS6 interacted with UBQLN1 (PLIC1), which interacts with many ubiquitin ligases and the proteasome^41^. UBQLN1 contains ubiquitin-like and ubiquitin-associated UBA domains and is implicated in protein traffic, autophagy, aggresomes, and cell spreading^42–44^. SOCS6 interacted with WDR40 and WDR28, which are deubiquitinase (DUB) activators^45, 46^. SOCS2 interacted with COPS2 and COPS3, components of the COP9 signalsome that controls CRL neddylation^47^. This was unexpected because the signalsome only binds to Neddylated CRLs^48^ and our samples were from cells that were incubated with the Neddylation inhibitor MLN4924^34^.

Both SOCS2 and SOCS6 also interacted with the POLR2A and POLR2H subunits of RNA polymerase II (PolII) and various PolII-associated TAFs. POLR2A is known to bind to the ElgB/C adaptor subunits of CRL5 through Elongin A (ElgA), using a SOCS box in ElgA^49, 50^. Our results suggest that SOCS2 and SOCS6 may interact with POLR2A-ElgA-ElgB/C, which is surprising because ElgB/C is only expected to bind one SOCS box protein at a time. SOCS2 and 6 also bound to the autophagy regulator, AMBRA1. AMBRA1 was reported to interact with ElgB/C and a variety of SOCS box proteins^51^. It is not known how ElgB/C binds to AMBRA1 at the same time as SOCS proteins.

We were most interested in proteins that may require phosphorylation in order to bind to SOCS2/6 SH2 domains. A previous screen for SOCS6 SH2-binding proteins detected insulin receptor substrate (IRS)-2 and 4 and the p85α and p85β subunits of phosphatidylinositol 3′ kinase (PI3K)^52^. We detected IRS4 binding to SOCS2 and 6. Other SOCS-IRS family protein interactions have been reported, so SOCS-IRS binding may be quite general^52–54^. However, SOCS6 gene disruption does not affect insulin signaling in mice^52^. We also identified STAT5b using SOCS2 as bait. SOCS2 null mice have prolonged STAT5b signaling^55^. Our results suggest that SOCS2 may bind phospho-STAT5b to directly negatively regulate signaling.

Known phosphotyrosine proteins that we detected binding to SOCS6 include LIM domain proteins TES and PDLIM5 (ENH), both of which are involved in cell-cell and cell-matrix attachments^56–58^, as well as MLLT4 (afadin, AF6), a Ras/Rap-regulated protein which regulates cell-cell junctions^59^. Known tyrosine-phosphorylated proteins that bound SOCS2 include the apical membrane protein Shroom1^60, 61^, CTNNB1 (β-catenin), which regulates cell-cell junctions^62^, Crk, a signaling adaptor protein^63, 64^, Ezrin, a protein that cross-links the plasma membrane to the active cytoskeleton^65^, and the RTK EphA2.

Visual inspection of the SOCS2 and SOCS6 binding partners indicated that both proteins interact with a wide variety of integral membrane proteins and membrane trafficking proteins, including Rab1a, solute carrier family proteins SLC25A11, SLC25A10 and SLC25A6, SEC61 translocon component SEC61A1, Golgi secretory carrier family protein SCAMP3, and the vacuolar ATPase subunit ATP6V1E1. In addition, SOCS6 interacted with four different 14-3-3 family proteins (YWHAQ, YWHAG, YWHAH) and known 14-3-3 interactor A-Raf. Both SOCS proteins bound nuclear proteins and mitochondrial proteins. These interactions suggest that SOCS2 and 6 can enter a variety of cell compartments and interact with shared and private partners.

To validate some of our candidate SOCS2-interacting proteins in a different cell type, we repeated the BioID assay, transiently expressing Myc-BirA, Myc-BirA-SOCS6, Myc-BirA-SOCS2, and a SH2 domain-inactivating mutant, BirA-SOCS2^R73K^, in HeLa cells. Cell lysates were purified with streptavidin and probed for GAPDH as a negative control and Cul5 as a positive control (Supplementary Fig. S1). None of the BirA fusion proteins stimulated GAPDH biotinylation. All three BirA fusion proteins stimulated Cul5 biotinylation, as expected. If MLN4924 was omitted, the Cul5 band became a doublet of Neddylated and Un-Neddylated Cul5 (Supplementary Fig. S1). We then tested candidate SOCS2-interacting proteins that have been reported to be tyrosine phosphorylated, including Ezrin, Crk, IRS4, EphA2, and β-Catenin. All of these proteins were strongly biotinylated in cells expressing BirA-SOCS2^WT^ but not by BirA alone, BirA-SOCS2^R73K^ or BirA-SOCS6 (Supplementary Fig. S1). This suggests that they are in close proximity to SOCS2 and their interaction requires tyrosine phosphorylation.

### SOCS2 SH2 domain binds to EphA2 through autophosphorylation sites in the kinase domain

We chose the candidate CRL5^SOCS2^ substrate, EphA2, for further study. EphA2 is over-expressed in breast cancer cells and its over-expression correlates with decreased EfnA1 expression and loss of estrogen receptor expression^66–68^. Increased EphA2 expression in Her2-positive breast cancer patients correlates with decreased disease-free and overall survival^69^. Although several RTKs have been reported to interact with SOCS proteins, interaction between an Eph family receptor and a SOCS protein has not been reported previously.

**Figure 2:**
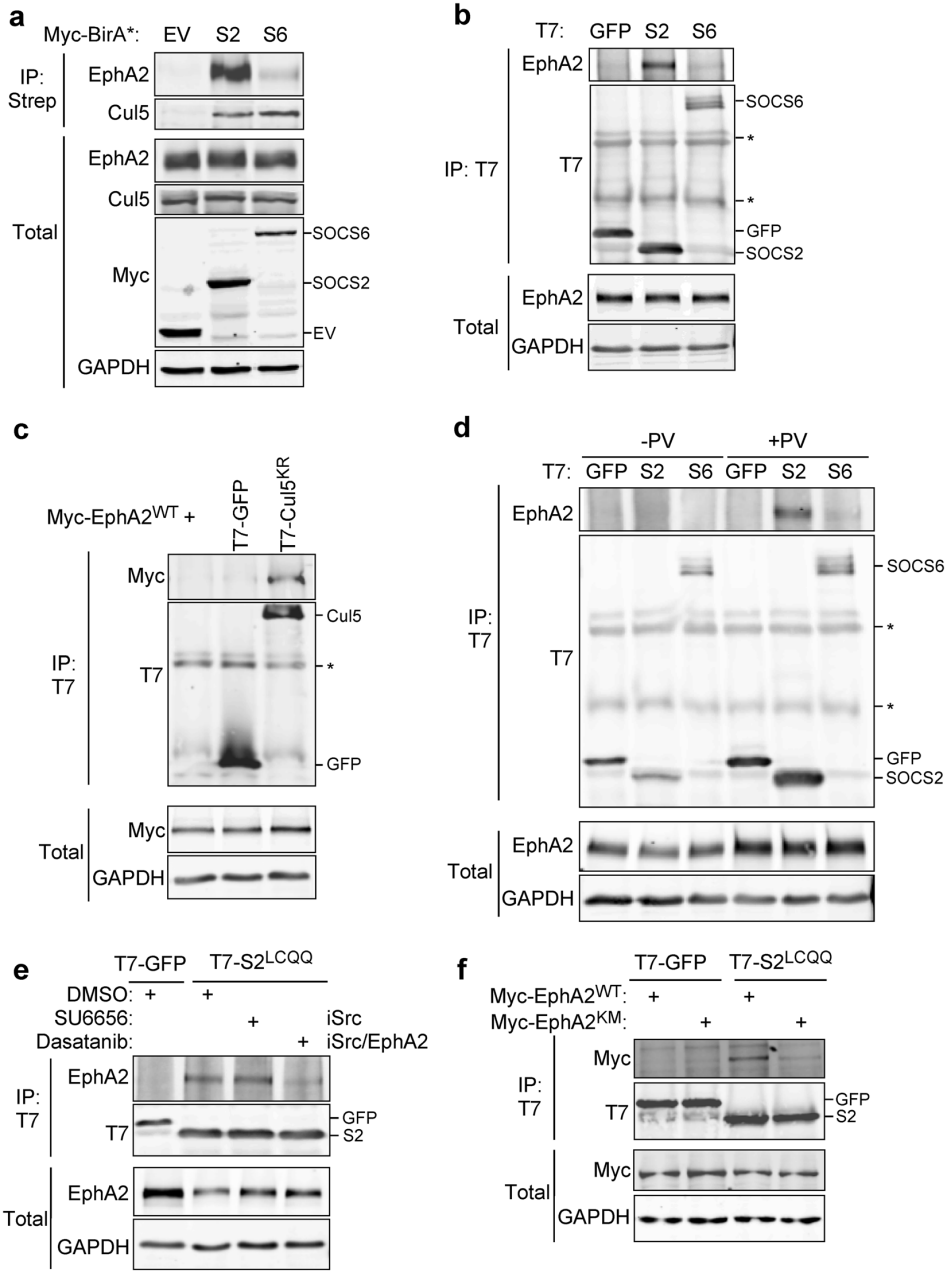
Endogenous EphA2 forms a complex with SOCS2 and CRL5, dependent on EphA2 kinase activity. (**a**) EphA2 is strongly biotinylated by BirA-SOCS2. 293T cells were transiently transfected with either Myc-BirA, Myc-BirA-SOCS2 or Myc-BirA-SOCS6. Twenty-four hours after transfection the cells were stimulated with 75 µM sodium orthovandate, 1 µM MLN4924 and 2 mM Biotin for twenty-four hours. Following the stimulation, the cells were lysed and biotinylated proteins were purified using streptavidin agarose beads. The whole cell extract represents 1.5% of the lysate used for the pull down. (**b**) EphA2 binds T7-SOCS2. HeLa cells were transiently transfected with T7-GFP, T7-SOCS2^LCQQ^, or T7-SOCS6^LCQQ^. Twenty-four hours later the cells were stimulated with 75 µM vanadate, 1 µM MLN4924 for twenty-four hours. The cells were lysed and immunoprecipitated with antibody to T7 and protein A/G beads. The whole cell extract represents 5% of the lysate used for the pull down. (**c**) EphA2 binds to inactive Cul5 (Cul5^K799R^). Cul5-depleted HeLa cells were transiently transfected with Myc-EphA2 and either T7-GFP or T7-Cul5^K799R^. Twenty-four hours after transfection the cells were stimulated with 1 mM pervanadate for 30 min, lysed and immunoprecipitated with antibody to T7 and protein A/G beads. The whole cell extract represents 6% of the lysate used for the pull down. (**d**) Omission of PTP inhibitors prevented co-precipitation of T7-SOCS2^LCQQ^ with EphA2. HeLa cells were transiently transfected with T7-GFP, T7-SOCS2^LCQQ^, or T7-SOCS6^LCQQ^. Twenty-four hours after the transfection half of the cells were stimulated with 75 µM sodium orthovandate or PBS for twenty-four hours. Cells were lysed and immunoprecipitated with antibody to T7 and protein A/G beads. The whole cell extract represents 7% of the lysate used for the pull down. (**e**) EphA2-SOCS2 interaction was inhibited by Dasatinib, but not by SU6656. HeLa cells were transiently transfected with T7-GFP or T7-SOCS2^LCQQ^. Twenty-four hours after the transfection either DMSO, 1 µM SU6656 or 200 nM Dasatinib were added to the cells for two hours, then stimulated with 1 mM pervanadate for 30 min, lysed and immunoprecipitated with antibody to T7 and protein A/G beads. The whole cell extract represents 3.5% of the lysate used for the pull down. (**f**) Kinase-dead EphA2 (K646M) cannot bind to T7-SOCS2^LCQQ^ in EphA2-depleted cells. EphA2-depleted HeLa cells were transiently transfected with either Myc-EphA2^WT^ or Myc-EphA2^K646M^ and either T7-GFP or T7-SOCS2^LCQQ^. Twenty-four hours after transfection the cells were stimulated with 1 mM pervanadate for 30 min, lysed and immunoprecipitated with antibody to T7 and protein A/G beads. The whole cell extract represents 3.5% of the lysate used for the pull down. Full-length blots are presented in Supplementary Fig. S7.

BioID validation experiments confirmed that EphA2 is in close proximity to BirA-SOCS2 (Fig. 2a). To determine if EphA2 forms a stable complex with SOCS2 or SOCS6, we tested for co-precipitation with T7-tagged SOCS box mutants, SOCS2^LCQQ^ and SOCS6^LCQQ^, which do not bind CRL5 and thus should not stimulate ubiquitylation and degradation^70^. Endogenous EphA2 co-precipitated with T7-SOCS2^LCQQ^ but not T7-SOCS6^LCQQ^ (Fig. 2b). These results suggest that EphA2 and SOCS2 are not only in close proximity but form a stable complex. To determine if Cul5 also bound EphA2, inactive Cul5 (T7-Cul5^K724R^) and Myc-EphA2 were co-expressed in Cul5-depleted HeLa cells. T7-Cul5^K724R^ co-precipitated Myc-EphA2 (Fig. 2c). This suggests that CRL5^SOCS2^ associates with EphA2 under these conditions.

In all of our previous experiments the phosphotyrosine phosphatase inhibitors orthovanadate or pervanadate were added to the cells prior to lysis. To determine if phosphatase inhibition was required for EphA2-SOCS2 binding, we immuoprecipitated T7-SOCS2 in the presence and absence of inhibitors. EphA2 did not co-precipitate with T7-SOCS2^LCQQ^ when phosphatases were active, providing evidence that the interaction may require tyrosine phosphorylation (Fig. 2d). EphA2 tyrosine phosphorylation may be catalyzed by EphA2, in an autophosphorylation reaction, or by cross-talk from another kinase. We used two approaches to test whether EphA2 ﻿kinase activity is required for SOCS2 binding. First, we tested whether chemical inhibition of EphA2 would inhibit SOCS2 binding. A specific inhibitor of EphA2 is not available, so we used Dasatinib, which inhibits EphA2, Src and several other kinases^71^. EphA2-SOCS2 interaction was inhibited by Dasatinib (Fig. 2e) but not by SU6656, a semi-specific Src inhibitor. Second, we utilized a kinase dead version of Myc-EphA2, EphA2^K646M^. When expressed in EphA2-depleted cells, Myc-EphA2^K646M^ was not phosphorylated on the Y588 autophosphorylation site and had decreased overall tyrosine phosphorylation (Supplementary Fig. S2). Myc-EphA2^K646M^ failed to bind to T7-SOCS2^LCQQ^ (Fig. 2f), suggesting that EphA2 autophosphorylation is required to bind SOCS2.

**Figure 3:**
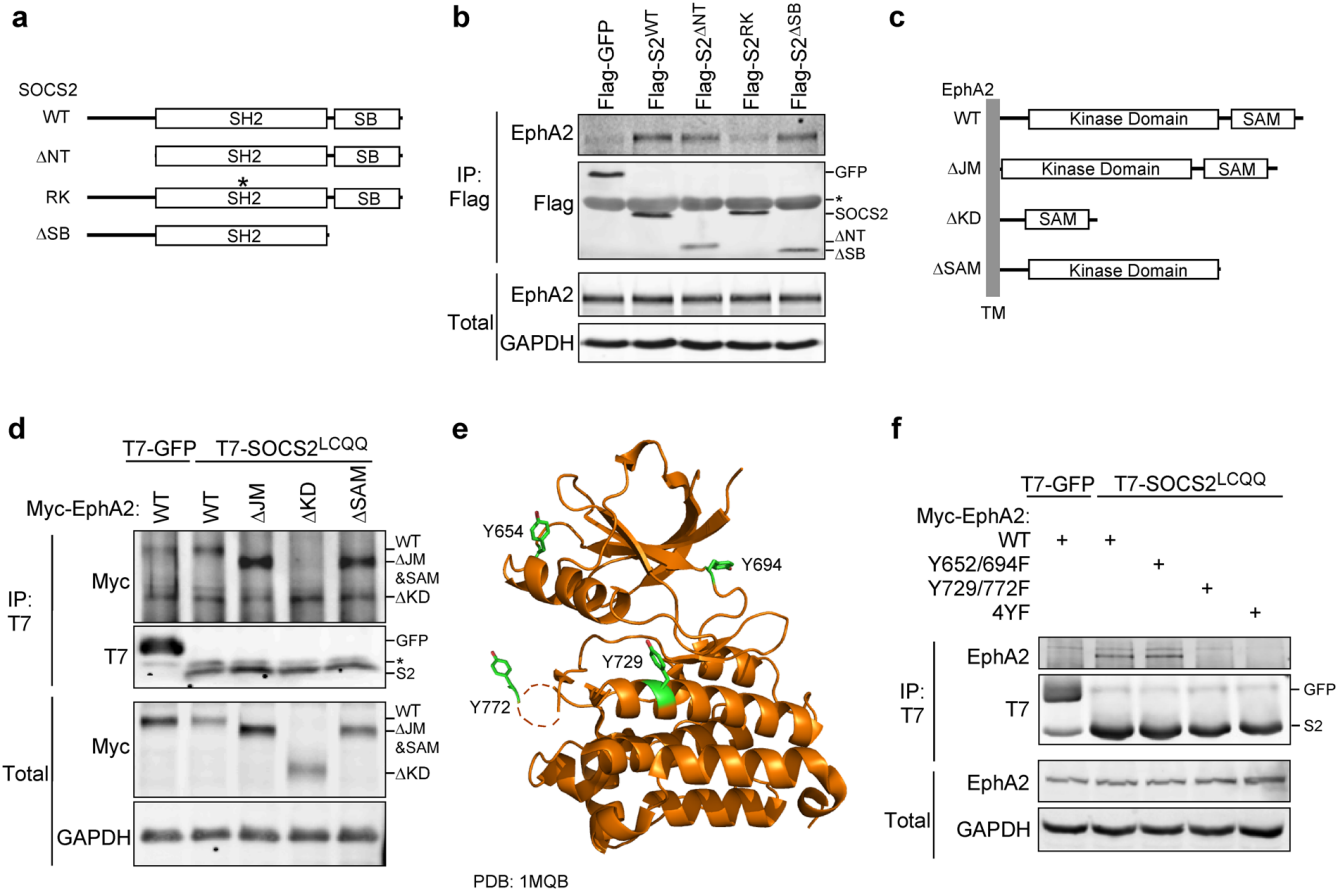
EphA2 Y772 and Y729 in the kinase domain bind to the SOCS2 SH2 domain. (**a**) Schematics of the SOCS2 deletion and point mutant constructs used: WT, full length SOCS2; ΔNT, lacking amino acids 1–37; RK, point mutation at R73 in the SH2 domain mutated to lysine; ΔSB, lacking amino acids 159–198. (**b**) The SOCS2 SH2 domain is required to bind EphA2. HeLa cells were transiently transfected with the indicated Flag-GFP or Flag-SOCS2 construct. Forty-eight hours after transfection the cells were stimulated with 1 mM pervanadate for 30 min, lysed and immunoprecipitated with Flag M2 magnetic beads. The whole cell extract represents 4% of the lysate used for the pull down. (**c**) Schematics depicting the cytosolic side of the EphA2 deletion mutants used: WT, full length EphA2; ΔJM, lacking amino acids 566–612; ΔKD, lacking amino acids 605–905; ΔSAM, lacking all amino acids after 886. (**d**) The EphA2 kinase domain is required for binding to SOCS2. HeLa cells were transiently transfected with T7-SOCS2^LCQQ^ and the indicated Myc-EphA2 construct. Forty-eight hours after transfection the cells were stimulated with 1 mM pervanadate for 30 min, lysed and immunoprecipitated with antibody to T7 and protein A/G beads. The whole cell extract represents 2% of the lysate used for the pull down. (**e**) Cartoon representation of the EphA2 kinase domain crystal structure (PDB: 1MQB)^104^, with Y654, Y694, Y729 and Y772 displayed as green sticks. (**f**) Y771 and Y729 in EphA2 are required for binding to SOCS2. 293T cells were transiently transfected with T7-SOCS2^LCQQ^ and either Myc-EphA2^WT^, Myc-EphA2^Y652F/Y694F^, Myc-EphA2^Y729F/Y772F^, and Myc-EphA2^Y652F/Y694F/Y729F/Y772F^. Twenty-four hours after transfection the cells were stimulated with 1 mM pervanadate for 30 min, lysed and immunoprecipitated with antibody to T7 and protein A/G beads. The whole cell extract represents 7% of the lysate used for the pull down. Full-length blots are presented in Supplementary Fig. ﻿S8.

To test whether EphA2-SOCS2 interaction involves the SH2 domain of SOCS2 we measured EphA2 binding to three SOCS2 mutants: an N-terminal deletion (ΔNT), an SH2 domain-inactivating mutant (R73K), and SOCS-Box deletion (ΔSB) (Fig. 3a). These mutants were transiently expressed in HeLa cells. The Flag-SOCS2^R73K^ mutant failed to interact with endogenous EphA2, while Flag-SOCS ΔNT and ΔSB both bound strongly to EphA2 (Fig. 3b). This suggests that the SOCS2 SH2 domain is binding to a phosphorylation site in EphA2.

The Eph receptor cytoplasmic region can be divided into four domains: a juxtamembrane segment, a kinase domain, a sterile alpha motif (SAM), and a PDZ-binding motif^72–74^. To narrow down the SOCS2 binding site in EphA2, we deleted the EphA2 juxtamembrane region (ΔJM), kinase domain (ΔKD), and SAM domain and PDZ-binding motif (ΔSAM) (Fig. 3c). T7-SOCS2^LCQQ^ bound to the Myc-EphA2^ΔJM^ and Myc-EphA2^ΔSAM^ but not to Myc-EphA2^ΔKD^ (Fig. 3d), suggesting that the SOCS2 binding site is located in the kinase domain of EphA2 and the JM and SAM regions are not required.

The EphA2 kinase domain contains eleven tyrosines. We mutated each of the eleven tyrosines individually to phenylalanine and tested binding to T7-SOCS2^LCQQ^ (Supplementary Fig. S3a–c). SOCS2 bound to each single mutant, so we tested the effects of double (Y652/694F & Y729/772F) and quadruple (4YF) mutations at four reported autophosphorylation sites^75^ (Fig. 3e). The Y729/772F and 4YF mutants both failed to interact while WT and Y652/694F EphA2 bound to T7-SOCS2^LCQQ^ (Fig. 3f). This suggests that both Y729 and Y772 are required for binding. Y772 is in the kinase activation loop, raising the possibility that the double mutant may have reduced autophosphorylation at multiple sites, leading to decreased binding. To exclude this possibility, we compared the phosphorylation of the single Y772F and double Y729/772F mutants (Supplementary Fig. S3d). Both mutants were phosphorylated similarly, but only the single Y772F mutant binds SOCS2, suggesting that both Y772 and Y729 are required for binding.

### EfnA1 induces EphA2 binding to SOCS2

**Figure 4:**
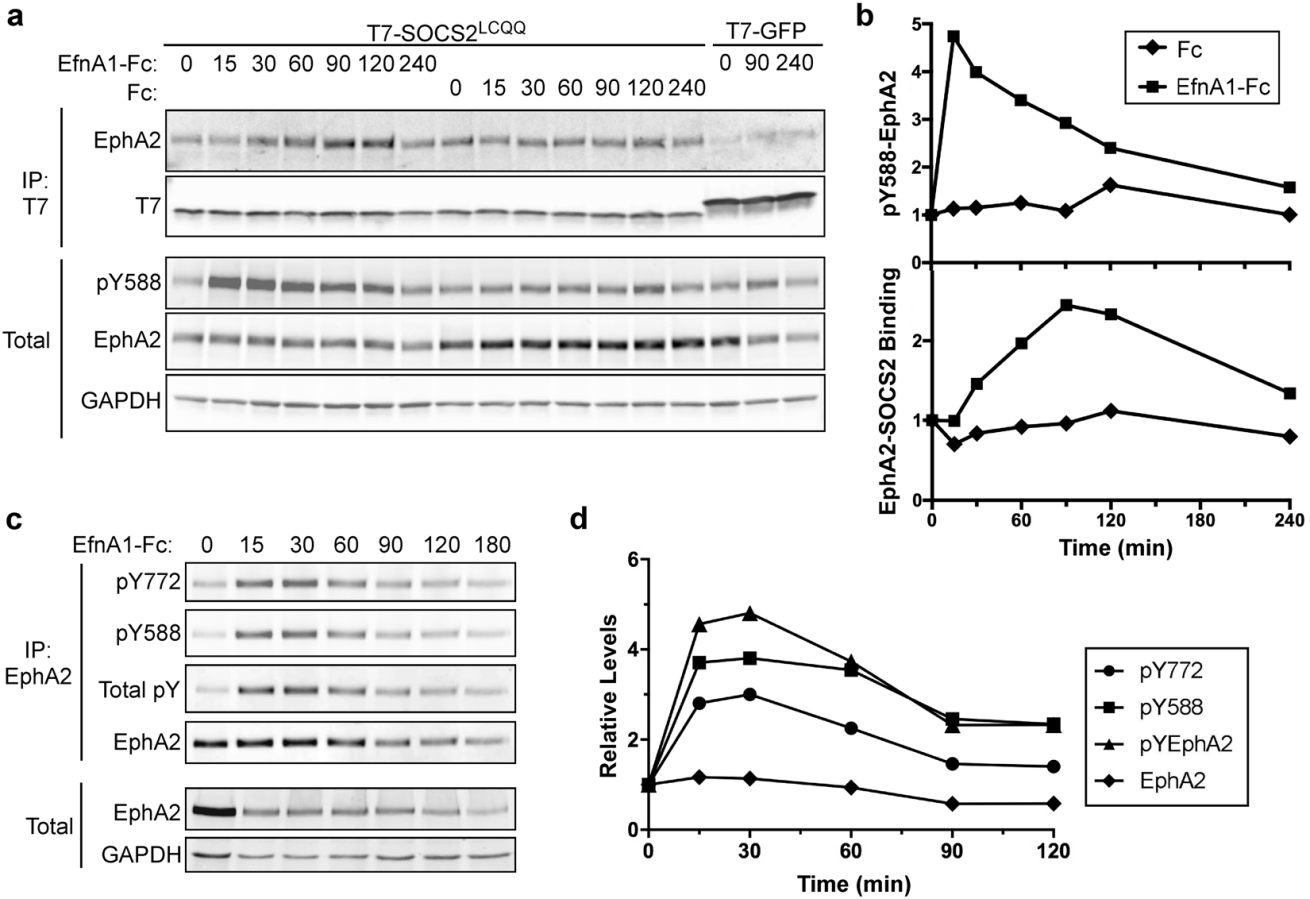
EphA2 activation by EfnA1 stimulates SOCS2-EphA2 binding after a delay. (**a**) EfnA1 stimulates EphA2 binding to SOCS2 and the binding kinetics lag behind EphA2 phosphorylation. HeLa cells were transiently transfected with T7- SOCS2^LCQQ^ or T7-GFP. Twenty-four hours after transfection the cells were starved in DMEM 0.5% BSA 10 mM HEPES for 4 hr. EfnA1-Fc or Fc (1 µg/mL) was added to cells, cells were then lysed at the indicated time, immunoprecipitated with antibody to T7 and then anti-mouse IgG Sepharose beads. The whole cell extract represents 6% of the lysate used for the pull down. (**b**) pY588-EphA2-peaks 15 min after EfnA-Fc addition and binding peaks 120 min after EfnA-Fc addition. Western blot images from Fig. 5a were analyzed using the ImageQuantTL software. Samples were normalized to time zero. (**c**) pY-EphA2, pY771, pY588 EphA2 display similar phosphorylation dynamics. HeLa cells were starved in DMEM 0.5% BSA 10 mM HEPES for 4 hr followed by 1 µg/mL EprinA1-Fc stimulation. Cells were lysed at the indicated time-points, immunoprecipitated with antibody to EphA2 and A/G agarose beads. The whole cell extract represents 12% of the lysate used for the pull down. (**d**) Quantification of EphA2 phosphorylation after EfnA1-Fc stimulation. Western blot images from the immunoprecipitation were analyzed using the ImageQuantTL software and normalized to time zero. Full-length blots are presented in Supplementary Fig. S9.

To test whether SOCS2 binds to EphA2 under physiological conditions, without phosphatase inhibitors, we stimulated cells with EfnA1-Fc, a dimer of EfnA1. HeLa cells were transfected with T7-SOCS2^LCQQ^, serum starved, and incubated with either EfnA1-Fc or with control Fc for various times. T7-SOCS2^LCQQ^ was immunoprecipitated and associated EphA2 was detected by immunoblotting. EfnA1-Fc but not Fc stimulated co-precipitation of EphA2 with SOCS2, with maximal binding 90–120 min after stimulation (Fig. 4a,b).

We tested whether SOCS association paralleled EphA2 phosphorylation by immunoblotting. Unfortunately, a phosphoepitope antibody to pY772 bound to a co-migrating protein; however, an antibody to the major juxtamembrane autophosphorylation site, pY588, was specific (Supplementary Fig. S4). Addition of EfnA1-Fc but not Fc stimulated pY588-EphA2 phosphorylation, with maximal phosphorylation 15–30 min after stimulation (Fig. 4a,b). Together this suggested that EfnA1 stimulates EphA2-SOCS2 association, but binding is delayed relative to EphA2 autophosphorylation at pY588.

We considered that EphA2-SOCS2 binding might be delayed because phosphorylation of the SOCS binding site, Y772, may also be delayed. The pY772 antibody is fairly specific for phosphorylation at Y772 when it is used on EphA2 immunoprecipitates (Supplementary Fig. S4). Therefore, we immunoprecipitated EphA2 at various times after EfnA1-Fc stimulation and probed immunoblots with antibodies to pY772, pY588 and total phosphotyrosine (Fig. 4c). Phosphorylation of all sites peaked around 15–30 min (Fig. 4d). Taken together, these results indicate that SOCS2 binding is increasing at the same time as pY772 phosphorylation is decreasing. This was surprising, and suggested additional regulation of EphA2-SOCS2 interaction.

### SOCS2 associates with internalized EphA2

**Figure 5:**
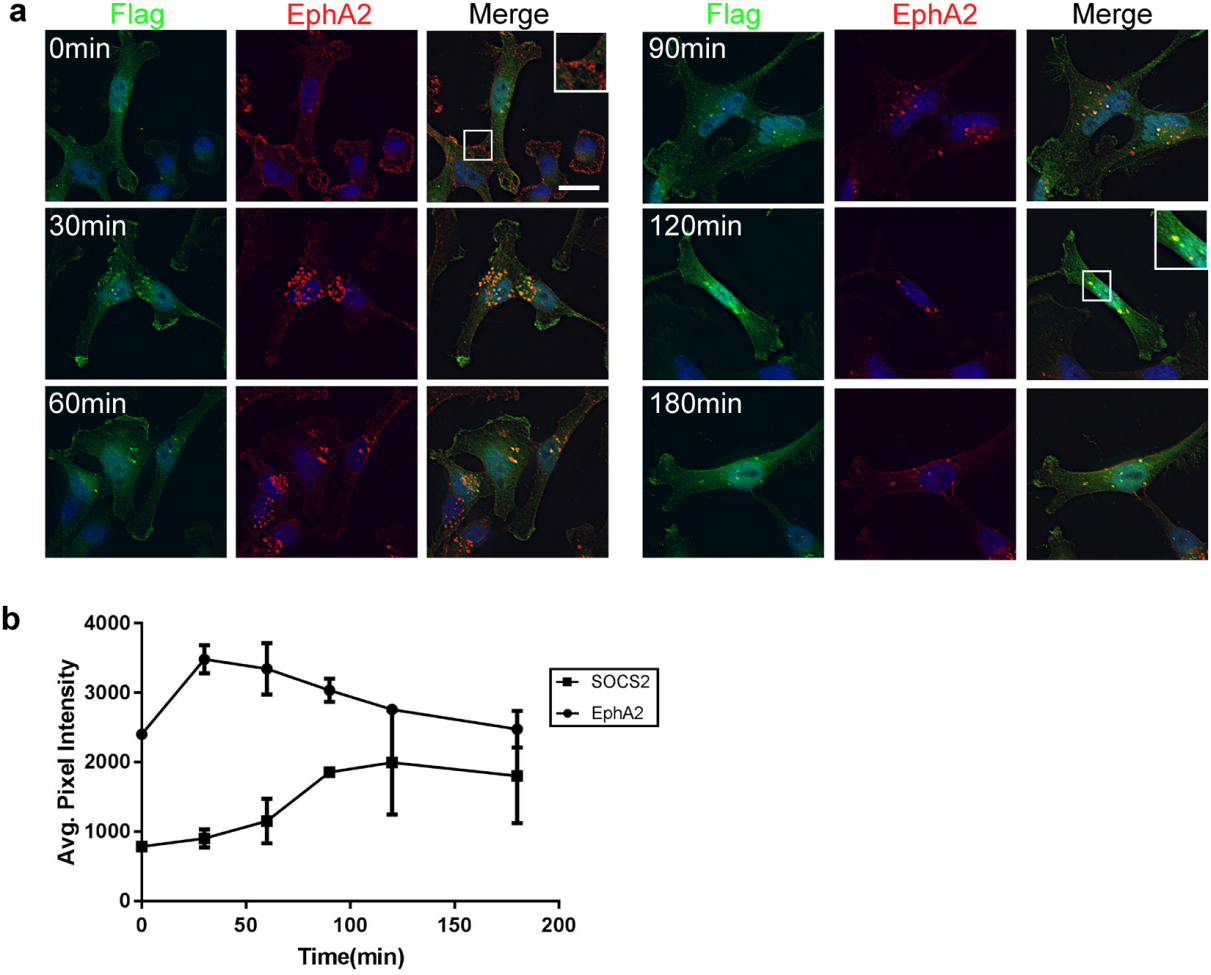
SOCS2 associates with internalized EphA2. (**a**) EfnA1 stimulates EphA2 internalization and association of SOCS2^LCQQ^ with endosomes containing EphA2. Flag-HA-SOCS2^LCQQ^ was stably expressed in MDA-MB-231 cells. Cells were starved in DMEM 0.5% BSA 10 mM HEPES for 4 hr followed by a 1 hr incubation with EfnA1-Fc (1 µg/mL) on ice. The ligand was washed off and the cells were placed at 37 °C for the indicated amount of time before being fixed. Fixed and permeabilized cells were stained with anti-Flag M2, anti-EphA2 and appropriate secondary antibodies. Images are a single Z-section. Exposure and brightness/contrast are the same for all pictures. Scale bar: 20 μm. (**b**) EphA2-SOCS2 co-localization is maximal 120 min after EfnA1-Fc addition. EphA2 vesicles were identified using the find object function in the Volocity Software. The intensities of EphA2 (circles) and SOCS2 (square) in these vesicles were measured. Points are mean and standard error of the mean of two biological independent experiments.

Efn’s activate Eph’s at the cell surface and then induce receptor internalization^76^. We wondered whether the delayed binding of SOCS2 to EphA2 reflects a dependence on EphA2 internalization. We followed EphA2 internalization using MDA-MB-231 cells, which have high levels of EphA2 and low levels of EfnA1 (Supplementary Fig. S5a). Flag-HA-SOCS2^LCQQ^ was stably expressed in these cells. To inhibit internalization and synchronize receptor traffic, cells were pre-incubated with EfnA1-Fc in the cold for 1 hr. Under these conditions, EphA2 was mostly on the plasma membrane and Flag-HA-SOCS2^LCQQ^ was diffusely distributed in the cytoplasm (Fig. 5a). Unbound EfnA1-Fc was then washed off and the cells were rapidly warmed to 37 °C to induce internalization. EphA2 rapidly translocated into intracellular vesicles, where it co-localized extensively with SOCS2 (Fig. 5a). Co-localization was maximal 30 to 120 min after warming (Fig. 5b). Immunoblotting showed that the receptor becomes phosphorylated during the pre-incubation with EfnA1-Fc at 4 °C and is progressively dephosphorylated after warming (Supplementary Fig. S5b). These results suggest that SOCS2 does not bind to active EphA2 at the plasma membrane and only associates with EphA2 after internalization, when EphA2 phosphorylation is declining.

**Figure 6:**
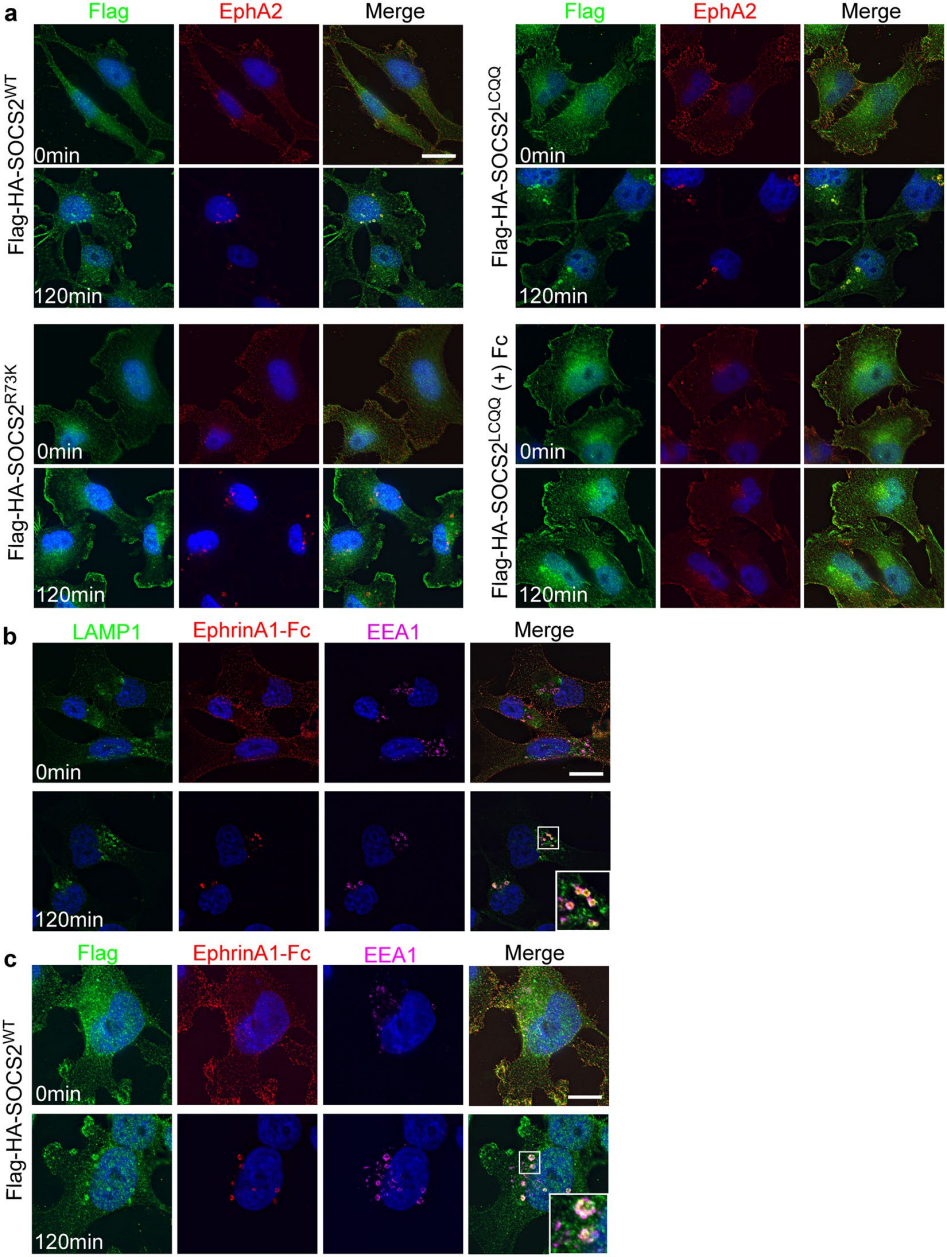
Requirement for SOCS2-EphA2 co-localization. (**a**) SOCS2-EphA2 co-localization on endosomes requires EfnA1 stimulation and SOCS2-EphA2 interaction, but not SOCS2-CRL5 interaction. Flag-HA-SOCS2^WT^ and Flag-HA-SOCS2^LCQQ^ but not Flag-HA-SOCS2^R73K^ ﻿form﻿﻿﻿ puncta 120 min after stimulation with EfnA1-Fc but not Fc. The Flag-HA-SOCS2 puncta co-localize with EfnA1-Fc. The indicated Flag-HA-SOCS2 constructs were stably integrated into MDA-MB-231 cells. Cells were starved in DMEM 0.5% BSA 10 mM HEPES for 4 hr followed by a 1 hr incubation with EfnA1-Fc or Fc (1 µg/mL) on ice. The ligand was washed off and the cells were placed at 37 °C for the indicated times and then fixed. Fixed and permeabilized cells were stained with anti-Flag M2, anti-EphA2 and appropriate secondary antibodies. Scale bar: 25 μm. (**b**) Endosomes that contain EfnA1-Fc are associated with LAMP1 and EEA1. Cells were starved and stimulated as in (**a**). Fixed and permeabilized cells were stained with anti-LAMP1, anti-Fc, anti-EEA1. Scale bar: 15 μm. (**c**) SOCS2 associates with EEA1-positive endosomes that contain EfnA1-Fc. Cells were starved and stimulated as in (**a**). Fixed and permeabilized cells were stained with anti-Flag M2, anti-Fc, anti-EEA1. Scale bar: 15 μm. In all cases, images are maximum intensity projections of 3 Z-sections. The scaled intensity is unequal in some images to allow for visualization. See methods for details.

We next tested whether SOCS2 relocalization required EphA2 activation and the SOCS2 SH2 domain. SOCS2 did not localize to endosomes in cells stimulated with Fc, suggesting that EphA2 activation is required for relocalization (Fig. 6a, Flag-HA-SOCS2^LCQQ^ + Fc). Relocalization did not require CRL5 binding (compare Flag-HA-SOCS2^WT^ and Flag-HA-SOCS2^LCQQ^, Fig. 6a). However, SH2 domain mutant Flag-HA-SOCS2^R73K^ did not re-localize, suggesting that SH2 domain-phosphotyrosine interaction is involved (Fig. 6a). The vesicles that contain EphA2 at 120 min also contain EfnA1-Fc, suggesting that the ligand has not been degraded (Supplementary Fig. S6). The SOCS2/EphA2/EfnA1-Fc vesicles are surrounded by the endosomal markers EEA1 and LAMP1 (Fig. 6b and c). This suggests that EphA2 receptor and EfnA1-Fc ligand are internalized and remain together, and the receptor traffics through the endosomal system where it associates with SOCS2. The presence of both EEA1 and LAMP1 on SOCS2/EphA2/EfnA1-Fc vesicles was surprising because they mark early and late endosomes, respectively^77, 78^. However, EphA2 is degraded slowly after EfnA1-Fc stimulation^71, 79, 80^, so these endosomes may be still maturing from early endosomes into multi-vesicular bodies. Altogether, these results suggest that EfnA1-Fc stimulation induces EphA2 phosphorylation and internalization, and SOCS2 only binds to autophosphorylated EphA2 after internalization to endosomes.

### SOCS2 overexpression induces EfnA1 expression and down regulates EphA2

**Figure 7:**
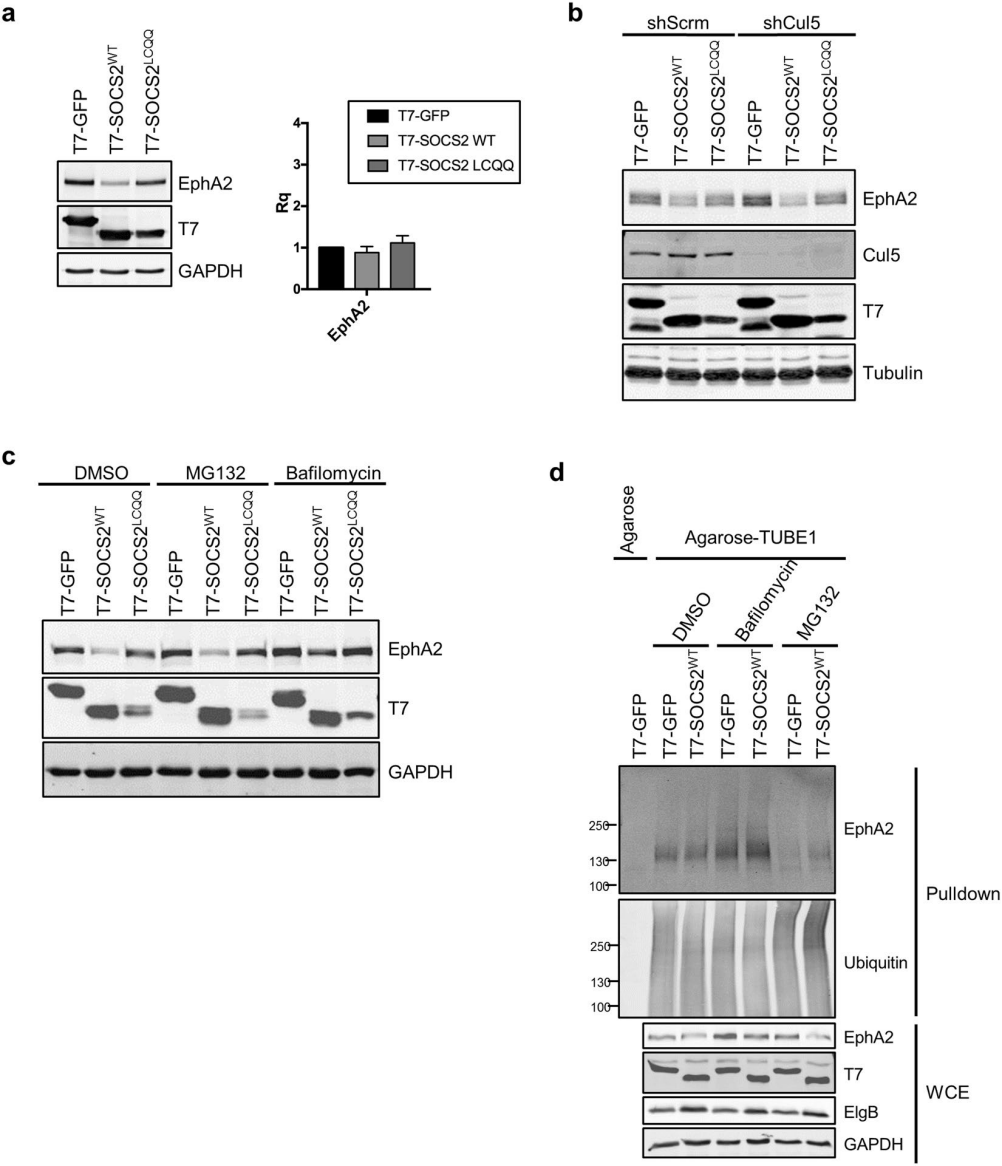
SOCS2^WT^ over-expression down-regulates EphA2 via the lysosome. (**a**) SOCS2^WT^ but not SOCS2^LCQQ^ overexpression decreases EphA2 protein level. HeLa cells were transiently transfected with ElgB/C and either T7-GFP, T7-SOCS2^WT^, or T7-SOCS2^LCQQ^. Total protein or RNA was harvested from the cells 48 hr after transfection. Proteins were analyzed by Western blotting. RNA was quantified using the ΔΔCt method with SYBR Green qPCR. Bars are mean and standard deviation of three independent experiments. (**b**) SOCS2 over-expression stimulates EphA2 down-regulation independent of CRL5. Control or Cul5-depleted HeLa cells were transiently transfected with ElgB/C and either T7-GFP, T7-SOCS2^WT^, or T7-SOCS2^LCQQ^. Forty-eight hours after transfection cell lysate was harvested and analyzed by Western blotting. (**c**) Lysosomal but not proteasomal inhibitors block the SOCS2-induced decrease in EphA2. HeLa cells were transiently transfected with ElgB/C and either T7-GFP, T7-SOCS2^WT^, or T7-SOCS2^LCQQ^. Forty-eight hours after transfection, DMSO, Bafilomycin or MG132 was added to the cells for a further 24 hr before lysis and analysis by Western blotting. (**d**) SOCS2 overexpression stimulates EphA2 ubiquitylation, that is further increased by lysosome, but not proteasome, inhibitors. HeLa cells were transiently transfected with ElgB/C and either T7-GFP, T7-SOCS2^WT^, or T7-SOCS2^LCQQ^. Forty-eight hours after transfection, DMSO, Bafilomycin or MG132 was added to the cells for 4 hr before lysis. Lysates were incubated with agarose or agarose-TUBE1 beads and ubiquitylated proteins were analyzed by Western blotting. The whole cell extract represents 2% of the lysate used for streptavidin pull down. Full-length blots are presented in Supplementary Fig. 10.

The delayed binding of SOCS2 to EphA2 at late endosomes led us to hypothesize that CRL5^SOCS2^ may catalyze EphA2 ubiquitylation after it has been internalized, providing a signal for incorporation into intraluminal vesicles and targeting for lysosomal degradation. To test whether SOCS2 regulates EphA2 degradation, we measured endogenous EphA2 levels in cells co-overexpressing SOCS2 and ElgB/C, which stabilizes SOCS2^81^. Over-expressed SOCS2^WT^ decreased EphA2 protein but not RNA, suggesting that degradation may be increased (Fig. 7a). To test whether SOCS2-induced EphA2 down-regulation involved CRL5, we over-expressed SOCS2^LCQQ^, which does not interact with CRL5. This mutant was less effective than SOCS2^WT^ in down-regulating EphA2 (Fig. 7a), but it was also under-expressed relative to SOCS2^WT^, presumably because SOCS2^LCQQ^ is not stabilized by ElgB/C^70^. As an alternative approach, we tested whether SOCS2^WT^ would induce EphA2 down-regulation in Cul5-depleted cells. EphA2 levels declined when SOCS2^WT^ was expressed even when Cul5 was absent (Fig. 7b). This suggests that SOCS2 targets EphA2 for degradation independent of CRL5.

To understand the mechanism by which SOCS2 down-regulates EphA2, we tested whether SOCS2-induced EphA2 down-regulation is inhibited by proteasomal or lysosomal inhibitors, MG132 and bafilomycin, respectively. In both control cells and cells over-expressing SOCS2, EphA2 levels increased when the lysosome but not proteasome was inhibited, suggesting that SOCS2 increases the basal rate of lysosomal degradation of EphA2 (Fig. 7c). To determine if SOCS2 stimulates EphA2 ubiquitylation, we isolated ubiquitylated proteins using Tandem Ubiquitin Binding Entities (TUBEs)^82^. Total protein ubiquitylation was not affected by lysosomal or proteasomal inhibitors or SOCS2 over-expression, so any differences in EphA2 abundance in the pulldowns can be attributed to changes in EphA2 ubiquitylation state (Fig. 7d). EphA2 ubiquitylation was increased by bafilomycin, consistent with destruction of ubiquitylated EphA2 by the lysosome. EphA2 ubiquitylation increased further when SOCS2^WT^ was over-expressed, despite a decrease in total EphA2 levels (Fig. 7d). Curiously, EphA2 ubiquitylation was reduced by MG132, potentially because MG132 depletes the free ubiquitin pool available for K63-linked ubiquitylation or multi-monoubiquitylation of proteins destined for lysosomal degradation^20, 83^. These results suggest two things: first, that EphA2 is constitutively ubiquitylated and degraded by the lysosome, and second, that SOCS2^WT^ overexpression stimulates muti-mono or polyubiquitylation and lysosomal degradation of EphA2.

**Figure 8:**
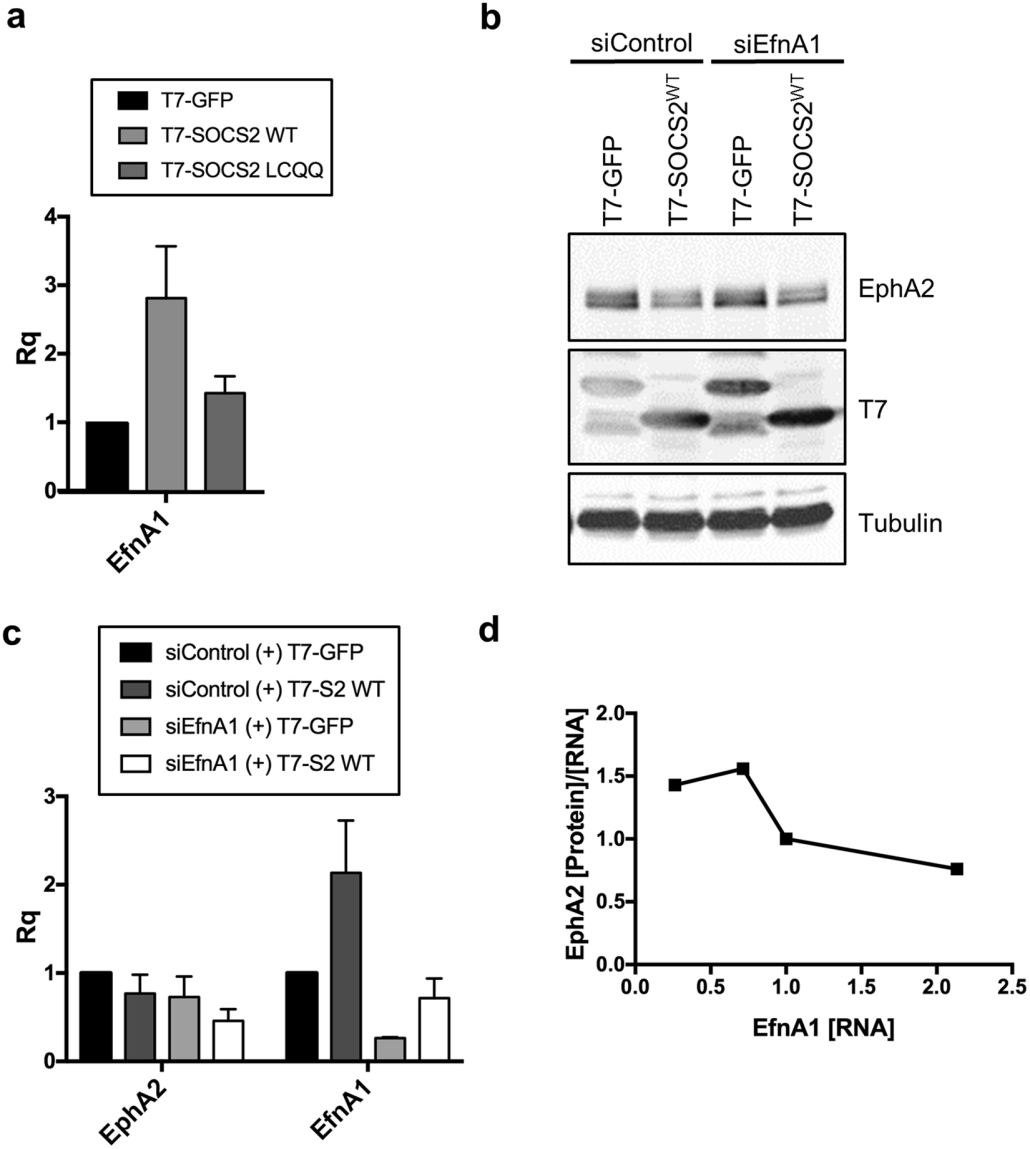
Endogenous EnfA1 promotes turnover of EphA2. (**a**) SOCS2^WT^ but not SOCS2^LCQQ^ overexpression induces EfnA1 expression. HeLa cells were transiently transfected with ElgB/C and either T7-GFP, T7-SOCS2^WT^, or T7-SOCS2^LCQQ^. Total protein or RNA was harvested from the cells 48 hr after transfection. Proteins were analyzed by Western blotting. RNA was quantified using the ΔΔCt method with SYBR Green qPCR. Bars are mean and standard deviation of three independent experiments. These are the same RNA samples analyzed in Fig. 7a. (**b**–**d**) EfnA1-depletion inhibits EphA2 down-regulation in cells overexpressing SOCS2^WT^. HeLa cells were transfected with the indicated siRNA, allowed to recover for 36 hr, then transfected again with the same siRNA. One day later, the cells were transfected with ElgB/C and either T7-GFP or T7-SOCS2^WT^. Forty-eight hours later, total protein or RNA was harvested. (**a**) Protein was analyzed by Western blotting. (**b**) RNA was quantified using SYBR Green qPCR and the ΔΔCt method. Bars are mean and standard deviation of two biological independent experiments. (**c**) EphA2 protein to RNA ratio is inversely correlated with the amount of EfnA1 RNA. Full-length blots are presented in Supplementary Fig. S﻿11.

SOCS2 may induce EphA2 turnover by a variety of indirect mechanisms. One possibility is that SOCS2 might increase production of EfnA1, thereby stimulating EphA2 lysosomal degradation. To assay EfnA1 gene expression, we measured EfnA1 mRNA levels in control and SOCS2 over-expressing cells. Remarkably, EfnA1 mRNA was strongly induced by SOCS2 over-expression (Fig. 8a). This suggests that SOCS2 may down-regulate EphA2 indirectly, by inducing EfnA1 and stimulating EphA2 lysosomal degradation. To check if EfnA1 is required for SOCS2-induced EphA2 down-regulation, we knocked down EfnA1 in control and SOCS2-expressing cells. Indeed, the EphA2 protein to RNA ratio was negatively correlated with the level of EfnA1 expression (Fig. 8b–d). Taken together, these results are consistent with a model in which SOCS2 over-expression induces EfnA1 gene expression, and autocrine stimulation by EfnA1 increases ubiquitylation and lysosomal turnover of EphA2, potentially by CRL5^SOCS2^-independent mechanisms.

## Discussion

SOCS proteins inhibit phosphotyrosine signaling in immune cells by several mechanisms, including directly inhibiting tyrosine kinases, masking phosphorylation sites, and recruiting CRL5 to ubiquitylate and ultimately degrade phosphorylated targets^84^. To gain insights into SOCS functions in non-immune cells, we sought new SOCS binding proteins by performing the first large scale affinity enrichment proteomics screens of SH2 domain-containing SOCS proteins. We detected 48 proteins that interacted with SOCS2 and 74 proteins that interacted with SOCS6. We found known interaction partners, including proteins involved in ubiquitylation or the proteasome, the CRL5 complex, and RNA polymerase II. One unresolved mystery is how SOCS2 and SOCS6 bind to proteins such as RNA pol II, that have their own substrate receptors^50^. Of the new SOCS2/6-interacting proteins detected, only a few were annotated as tyrosine kinase substrates and few were clearly signaling proteins. Notably, both SOCS2 and 6 interacted with a variety of transmembrane proteins and vesicle traffic proteins, perhaps indicating a role for SOCS proteins in protein traffic.

We chose a set of SOCS2-interacting proteins previously reported to be tyrosine phosphorylated for functional validation. These proteins (Ezrin, Crk, IRS4, EphA2 and β-catenin) were confirmed to interact with SOCS2 and not SOCS6 in another cell type. Interactions were diminished when the SOCS2 SH2 domain was inactivated, suggesting that the interaction is mediated by phosphotyrosine. However, many of the other proteins we detected are not known to be tyrosine phosphorylated. Binding of these proteins may involve the divergent N-terminal regions of SOCS2 and SOCS6. SOCS6 in particular has a very long N-terminus (384 amino acids). Some of the non-phosphorylated proteins identified in our screen may bind to the N-termini of SOCS2 and SOCS6 and further study of the interactions will help elucidate the function of this region. The novel hits that we identified may help define additional biological roles for SOCS proteins in epithelial cell biology.

We focused on EphA2 as a representative of a group of tyrosine-phosphorylated proteins that interact with SOCS2 and each other (according to the STRING database), suggesting SOCS2 SH2-phosphotyrosine interactions. Indeed, EphA2-SOCS2 binding requires the SOCS2 SH2 domain, EphA2 autophosphorylation in the kinase domain, and two kinase domain tyrosine residues, Y772 and Y729. Y772 is the activation loop tyrosine - a well-known EphA2 autophosphorylation site. This region is disordered when EphA2 is active and may be accessible to the SOCS2 SH2 domain^85, 86^. Y729 was once identified as an autophosphorylation site^75^ but this may not be the case^87^. It is possible that Y729 plays a structural role in binding SOCS2. The results suggest that phosphorylation of Y772 by EphA2 creates a binding site for SOCS2, with contribution from Y729.

SOCS2 binding to EphA2 was stimulated by the EphA2 ligand EfnA1, suggesting it may be physiologically important. Curiously, however, EphA2-SOCS2 interaction did not parallel EphA2 phosphorylation. Rather, EphA2-SOCS2 binding lagged behind EphA2 phosphorylation by 30–60 min. Delayed binding of RTKs to SOCS proteins has been observed before. SOCS2 binds to IGF-IR 40 min after IGF-I stimulation^88^, which is 20 min after phosphorylation has peaked^89^. Kit ligand activates Kit phosphorylation at 5 min, but SOCS6 binding to Kit is maximal 30–60 min later^9^. This suggests that other factors may regulate RTK-SOCS binding in cells. For example, a SOCS protein may be unable to bind an RTK soon after activation due to masking of the binding site by other molecules. Alternatively, there may be delayed activation, recruitment or induction of other factors that are needed to stabilize the RTK-SOCS interaction. In the case of SOCS2 and EphA2, we found that delayed binding may reflect a requirement for ligand-stimulated EphA2 internalization. SOCS2 and EphA2 did not co-localize significantly at the cell surface, but co-localized on endosomes after EfnA1-Fc stimulation. These endosomes persisted for up to 2 hr and contained EfnA1-Fc and EphA2 together with EEA1 and LAMP1, markers for early and late endosomes. This suggests that SOCS2-EphA2 association may be blocked at the plasma membrane or stimulated on endosomes. If the latter, then SOCS2 binding to EphA2 may be stabilized by secondary interactions between SOCS2 and endosomal membranes, perhaps through binding interactions we detected with endosomal proteins such as the vacuolar ATPase and SCAMP3. There may also be avidity effects: EphA2 is much more concentrated on endosomal membranes after internalization than on the plasma membrane, and the increased density of EphA2 in endosomes may decrease the SOCS2 dissociation rate and increase the effective avidity.

EphA2 undergoes constitutive endocytosis and rapid recycling back to the surface regardless of ligand stimulation^90^. This constitutively internalized EphA2 may be spontaneously activated at the surface, perhaps by crowding, and is dephosphorylated in Rab11-positive endosomes before recycling back to the surface^79, 90^. When ligand is added, some of the internalized EphA2 is diverted to the lysosome^91^. We found that inhibiting the lysosome but not the proteasome increased ubiquitylation of EphA2 and the total level of EphA2, suggesting that a portion of EphA2 is constitutively targeted to the lysosome for degradation. Since HeLa cells express endogenous EfnA1, this constitutive turnover may be due to autocrine activation of EphA2 by EfnA1 made in the same cell or neighboring cells. When SOCS2 was over-expressed, endogenous EfnA1 production increased, as did the ubiquitylation and lysosomal degradation of EphA2. Indeed, SOCS2-induced down-regulation of EphA2 required EfnA1. Moreover, the SOCS2-stimulated degradation of EphA2 was Cul5-independent. This suggests that the effect of over-expressed SOCS2 on EphA2 is quite indirect, involving induction of EfnA1 and increased EfnA1-induced internalization and degradation of EphA2.

EphA2 has been reported to bind to two additional E3 ubiquitin ligases: Cbl and UBE4A-SLAP. Cbl binds to activated EphA2 and Cbl over-expression increases ubiquitylation and degradation of over-expressed EphA2^79, 92, 93^. The adaptor protein Src-like adaptor protein (SLAP) binds to Y594 in the EphA2 juxtamembrane region, which then recruits to the E3 Ub ligase UBE4A^94^. UBE4A-SLAP has been shown to ubiquitylate EphA2 in cells and *in vitro*, and knockdown of SLAP or of UBE4A slightly increased total EphA2 protein in colorectal cancer cell lines. Cbl or SLAP-UBE4A may be responsible for the increased turnover of EphA2 in SOCS2-overexpressing cells.

Ligand activation of other RTKs stimulates binding to various E3s, including Cbl, as well as SOCS proteins. For example insulin stimulates INSR-SOCS1,3,6; Flt ligand stimulates Flt3-SOCS1,2,6; EGF stimulates EGFR-SOCS2,3,4,5,6; and SCF stimulates Kit-SOCS1,4,6^8–10, 12–18, 95–97^. However, all of these SOCS-RTK interactions have been shown using exogenous expression of the SOCS protein and the biological/mechanistic links between SOCS and RTKs have been elucidated through SOCS overexpression studies. Additionally, none of these studies checked mRNA expression of the RTK or their associated ligand(s). Our results suggest that SOCS overexpression may result in indirect effects on RTK ligand expression and this can provide a biological/mechanistic link between SOCS and RTKs. If SOCS overexpression increases ligand expression for other RTKs, then the indirect effects of increased ligand expression will need to be separated from the direct effect of SOCS-CRL5 down regulating the RTK protein. The key experiment will be to perform loss of function studies on these SOCS-RTK interactions to confirm conclusions made with exogenously expressed SOCS. Either way there is a need for more studies on RTK-SOCS interactions to confirm the mechanism and biological roles of these interactions.

## Materials and Methods

### Plasmids

pCAG-T7-mCul5^K799R^, pCAG-T7-mSOCS2 and pCAG-T7-mSOCS6 constructs have been previously described^27, 98^. The T7-mSOCS2^LCQQ^ and T7-mSOCS6^LCQQ^ mutants were made using PfuTurbo DNA Polymerase to perform site directed mutagenesis followed by DpnI digestion. The pCAG-ElgC-T2A-ElgB was described earlier^98^.

pHAGE-Flag-HA-hSOCS2^WT^ and pHAGE-Flag-HA-hSOCS2^LCQQ^ plasmids were made as follows. hSOCS2 was PCR amplified using Phusion taq polymerase from MCF10A cDNA by Sashcha Straight. The hSOCS2 PCR product was then PCR amplified using PfuTurbo with flanking attB sties. The attBhSOCS2 PCR product was moved it into the pDONR221 vector using a BP reaction. The pDONR221-hSOCS2^LCQQ^ was made using PfuTurbo DNA polymerase to perform site directed mutagenesis followed by DpnI digestion. The pDONR221-hSOCS2^WT^ and hSOCS2^LCQQ^ were then recombined into the Gateway destination vector pHAGE-N-Flag-HA-IRES-PURO, a kind gift from Wade Harper^99^, using a LR reaction. The pHAGE-Flag-HA-hSOCS2^R73K^ was made by mutating pHAGE-Flag-HA-hSOCS2^WT^ using PfuTurbo DNA polymerase to perform site directed mutagenesis followed by DpnI digestion.

The pcDNA3.1-Myc-BirA-mSOCS2 and pcDNA3.1-Myc-BriA-mSOCS6 vectors were made as follows. The pcDNA3.1-Myc-BirA was purchased through Addgene (35700). The T7-mSOCS2 and T7-mSOCS6 were PCR amplified with 5′ EcoRI and 3′ KplI flanking sites from pCAG-T7-mSOCS2 and mSOCS6 using PfuTurbo. The PCR products were then subcloned into pcDNA3.1-Myc-BirA vector downstream of BirA.

The pcDNA5-Myc-BirA FRT/TO, pcDNA5-Myc-BirA-T7-mSOCS2 FRT/TO and pcDNA5-Myc-BirA-T7-mSOCS6 FRT/TO vectors were made as follows. The pcDNA5/FRT/TO vector (V6520202) was purchased from Thermo Fisher Scientific. A unique NheI site was introduced into the polylinker of the pcDNA5/FRT/TO vector. The Myc-BirA, Myc-BirA-T7-mSOCS2 and Myc-BirA-T7-mSOCS6 were PCR amplified with 5′-EcoRV and 3′-SpeI primers using Herculase II Fusion polymerase (Agilent) using the appropriate pcDNA3.1-Myc-BirA vector as a template. The PCR product was cut with EcoRV and SpeI and ligated into the altered pcDNA5/FRT/TO vector between EcoRV and NheI.

The pcDNA5-N-3xFlag-hSOCS2 FRT/TO and pcDNA5-N-3xFlag-mSOCS6 FRT/TO plasmids were made as follows. The mSOCS6 WT was PCR amplified with flanking attB sties from pCAG-T7-mSOCS6 using PfuTurbo and moved into the pDONR221 vector using a BP reaction. The destination vector pDEST-5′-TripleFlag pcDNA5 FRT TO with stop codon was a kind gift from Anne-Claude Gingras. The pDONR221-hSOCS2 and pDONR221-mSOCS6 were moved into pDEST-5′-TripleFlag pcDNA5 FRT TO using a LR reaction.

The Igk–Myc hEphA2 WT, hEphA2-ΔKD (aa 1–606 and 906–976), and hEphA2-ΔSAM (aa 1–886) were a kind gift from Hironori Katoh^100^. Igk–Myc hEphA2-ΔJM (aa 1–565 and 613–975) was made using PfuTurbo DNA Polymerase to perform site directed mutagenesis followed by DpnI digestion. The Igk–Myc EphA2 single amino acid mutants were made using PfuTurbo DNA polymerase to perform site directed mutagenesis followed by DpnI digestion.

pEF-BOS-FLAG-mSOCS2 WT, mSOCS2 ΔNT, and mSOCS2 ΔSB were a kind gift from Amilcar Flores-Morales^81^. The pEF-BOS-FLAG-mSOCS2 R73K was made using PfuTurbo DNA polymerase to perform site directed mutagenesis followed by DpnI digestion.

The pCAG-Flpe was purchased from Addgene (#13787). pOG44 was a kind gift from Anne-Claude Gingras.

pMXpuroII-shCul5 was described previously^101^. pLKO.1-nontarget small hairpin RNA (shRNA) control vector (SHC002) and pLKO.1-shEphA2 vector (TRCN0000006403) were purchased from (Sigma Aldrich).

### Cell lines

Flp-IN T-Rex 293 cells were purchased (Thermo Fisher Scientific) and grown in DMEM 10% FBS, 100 µg/mL Zeocin, 15 µg/mL Blasticidin. The Flp-IN T-Rex 293 cells were transfected with a corresponding FRT/TO vector and either pCAG-Flpe or pOG44 to flp in the gene of interest. The transfected cells were then selected in DMEM 10% Tet-Free FBS, 150 µg/mL Hygromycin B, 15 µg/mL Blasticidin and grown in this media from then on.

HeLa, 293T, and MDA-MB-231 cells were cultured in DMEM supplemented with 10% FBS and penicillin/streptomycin both at 100 U/ml. Recombinant lentiviruses containing shEphA2 were packaged using HEK 293T cells, and infections were carried out by standard protocols. After selection with 10 µg/ml puromycin, cells were maintained in the presence of antibiotic. Recombinant retroviruses containing pMXpuroII-shCRL5 were packaged using HEK 293T cells, and infections were carried out by standard protocols. The cells were then selected and grown in 10 µg/mL puromycin.

Recombinant lentiviruses containing pHAGE-Flag-HA-hSOCS2^WT^, hSOCS2^R73K^ and hSOCS2^LCQQ^ were packaged using HEK 293T cells, and infections were carried out by standard protocols. After selection with 5 μg/ml puromycin cells were maintained in the presence of antibiotic.

### Antibodies

The following antibodies were used: mouse anti-EEA1, rabbit anti-Crk (BD Biosciences), rabbit anti-ElgB, rabbit anti-GAPDH, rabbit anti-Cul5, mouse anti-Cul5, mouse anti-IRS4, mouse anti-Myc (9E10) and mouse anti-tubulin (Santa Cruz Biotechnology, Inc.), rabbit anti-beta catenin, mouse anti-Flag (M2) (Sigma-Aldrich), mouse anti-EphA2 (8B6), rabbit anti-EphA2 (D4A2), rabbit anti-EphA2 (pY588), rabbit anti-EphA2 (pY772), rabbit anti-EEA1, rabbit anti-Ezrin (Cell Signaling Technology); mouse anti-T7 (EMD Millipore), mouse anti-HA (HA.11) (BioLegend), rabbit anti-HA (Bethyl) and goat anti-human IgG Fc (Jackson ImmunoResearch Inc.). 4G10 was made in-house from the 4G10 hybridoma.

### qPCR of RNA Abundance

RNA was extracted using the Qiagen RNeasy Mini kit. cDNA was synthesized using a iScript kit (BioRad). The abundance of the cDNA was measured by qPCR using the iTaq Universal SYBR Green Supermix kit (BioRad), and run on the 7900HT Real Time PCR System or QuantStudio 5 Real Time (qPCR) System using the SDS software or QuantStudio respectively (Applied Biosystems & Thermo Fisher Scientific). The following primers were used:

EphA2 forward: 5′-CTGGTCTGCAAGGTGTCTGA-3′

EphA2 reverse: 5′-TTGGACAACTCCCAGTAGGG-3′

EphrinA1 forward: 5′-CACACCGTCTTCTGGAACAG-3′

EphrinA1 forward: 5′-CTCATGCTCCACCAGGTACA-3′

GAPDH forward: 5′-CAGCCTCAAGATCATCAGCA-3′

GAPDH reverse: 5′-TGTGGTCATGAGTCCTTCCA-3′

### DNA transfections, Cell Lysis, Western Analysis

HeLa cells were plated at 80% confluence in either a 6 cm or 6-well plates. A DNA/Lipofectamine 2000 mixture, made according to manufacture protocol, was added to the cells immediately after plating and left on the cells for 4 hr. Around 24 or 48 hr later cells were washed two times in phosphate-buffered saline (PBS) and lysed in X-100 buffer (1% Triton X-100, 150 mM NaCl, 10 mM HEPES (pH 7.4), 2 mM EDTA, 50 mM NaF) with fresh protease inhibitors (10 µg/mL Aprotinin, 10 µg/mL Leupeptin, 1 mM sodium vanadate, 1 mM PMSF).

Proteins were resolved by SDS-PAGE using a 30:1 acrylamide:bisacrylamide ratio and subsequently transferred onto nitrocellulose membrane. The membrane was blocked in Odyssey Blocking Buffer (LI-COR Biosciences-U.S.), for phosphotyrosine antibodies, or 5% non-fat dry milk in Tris-buffered saline with 0.1% Tween (all other antibodies), probed first with the indicated primary, followed by either IRDye 680RD goat anti-mouse or IRDye 800CW goat anti-rabbit conjugated secondary antibodies and visualized using the Odyssey Infrared Imaging System (LI-COR Biosciences-U.S.). Blot image display were scaled using the linear manual scaling in the LI-COR software and exported as an 8-bit gray scale image. Images were then imported into Canvas and brightness/contrast was adjusted. Figures 7 and 8 were assembled in PowerPoint, for these figures the brightness/contrast were adjusted in ImageJ prior to assembling in PowerPoint.

### Immunoprecipitation

Immunoprecipitation of EphA2 and T7- or Myc-tagged proteins was performed as described in ref. 27. The Flag immunoprecipitation was performed as previously described with the following changes: anti-Flag M2 magnetic beads (Sigma-Aldrich) were used instead of antibody and AG beads. Unless otherwise noted all immunoprecipitations were stimulated with 1 mM pervanadate for 30 min prior to cell lysis. Immunoprecipitation from cells stimulated with EphrinA1-Fc (R&D Systems) or hIgG1 Fc (Life Technologies) was done as follows. T7-tagged SOCS2 or GFP proteins were transfected into HeLa cells using Lipofectamine 2000. Twenty-four hours after transfection, cells were washed twice with PBS and starved in DMEM 0.5% BSA overnight. The following day cells were stimulated with either 1 µg/mL EphrinA1-Fc or Fc for the corresponding amount of time, lysed on ice in cold X-100 buffer containing fresh protease inhibitors, followed by centrifugation for 15 min at 14,000 × g. Lysates were rotated with mouse anti-T7 antibody for 2 hr at 4 °C. Anti-mouse IgG Sepharose Bead Conjugate (Cell Signaling Technology) was added to the lysate and antibody mix for 1 hr at 4 °C. After centrifugation, the protein–antibody–bead complex was washed three times in lysis buffer, re-suspended in Laemmli buffer, then resolved by SDS-PAGE.

### Immunofluorescence

MDA-MB-231 cells were plated on 12 mm glass coverslips, coated with 1 mg/mL poly-L-lysine (Sigma Aldrich) in borate buffer (40 mM boric acid, 19 mM sodium tetraborate, pH 8.5) for 24 hr. Cells were washed twice with PBS and starved in DMEM, 0.5% BSA, 10 mM HEPES for 4 hr. The cells were then moved onto wet ice, washed one time in ice cold PBS followed by the addition of DMEM 0.5% BSA, 10 mM HEPES containing 1 µg/mL EphrinA1-Fc or 1 µg/mL Fc. The plate was incubated with the ligand on wet ice at 4 °C for 1 hr. At the end of the hour the coverslips were washed three times with ice cold PBS, followed by the addition of 37 °C DMEM 0.5% BSA, 10 mM HEPES. The plate containing the coverslips was moved to a water bath in an incubator. Cells were fixed in 4% PFA in PBS at 4 °C for 30 minutes at each corresponding time point. Cells were then permeabilized with 0.1% Triton X-100 in PBS for 5 min at 25 °C, washed in PBS and blocked for 1 hr in 5% normal goat serum or 5% BSA in PBS before primary antibody was added for either for 1 hr at 25 °C or overnight at 4 °C. Coverslips were rinsed in PBS before the addition of Alexa Fluor 488-, Alexa Fluor 568- or Alexa Fluor 647-conjugated secondary antibodies, diluted 1:1,000, for 1 h at 25 °C. After several PBS rinses, coverslips were mounted in ProLong Gold with DAPI solution. For the stainings containing the anti-hFc antibody, a two-step staining protocol was followed to prevent cross reactivity with the rabbit primary. Briefly, cells were blocked, incubated with anti-hFc, Alexa Fluor 568- secondary, blocked, incubated with rabbit and mouse primary antibodies and finally with Alexa Fluor 488- and Alexa Fluor 647-conjugated secondary antibodies.

### Imaging

Fixed cells were visualized using a 40×, 60× or 100× numerical aperture (NA) 1.4 oil objective on a DeltaVision IX70 microscope (Olympus). Images were recorded using fixed camera settings (IX- HLSH100; Olympus). Images were acquired and deconvolved using softWoRx (Applied Precision, Issaquah, WA), and all exposure times were equal within an experiment. Deconvolved images from single plane in the cells or maximum intensity *z*-projections were used in the figures. Image scaling was equal for all images in a panel with the following exceptions: Fig. 7A 488 channel upper contrast, 594 upper contrast; Fig. 7B 488 channel upper contrast, 594 upper contrast; Fig. 7C 488 upper and lower contrast and 594 upper contrast. Figures were assembled using Canvas (ACD Systems) software.

### Image Analysis

To quantify co-localization between EphA2 and Flag-HA-SOCS2^LCQQ^ entire z-sections were moved into Volocity software (PerkinElmer) and EphA2 vesicles were identified using the find object function with these settings (Intensity = 1750-Max and Size >0.2 µm). The mean EphA2 and Flag intensity in these objects are reported on the graphs.

### Triple Flag Affinity Purification

For the FLAG pull-downs, 2 × 150 cm^2^ dishes of 3xFLAG, 3xFlag-SOCS2 or 3xFLAG-SOCS6 293 T-REx cells were grown to 80% confluence then treated with 75 µM vanadate, 1 µM MLN4924, 1 µg/mL Doxycycline for 24 hr. They were then washed twice in 20 mL PBS, scraped into PBS, pooled and collected by centrifugation at 5,000 × *g* for 5 min at 4 °C. Cell pellets were snap frozen in dry ice and stored at −80 °C until lysis. The cell pellet was weighed and 1:4 pellet weight/lysis buffer (by volume) was added. Lysis buffer consisted of 50 mM HEPES-KOH (pH 8.0), 100 mM KCl, 2 mM EDTA, 0.1% Nonidet P-40, 10% glycerol, 1 mM PMSF, 1 mM DTT and 1x protease inhibitor mixture tablet (Sigma-Aldrich). Upon addition of lysis buffer, cells were incubated in a 37 °C water bath with continuous agitation until the pellet melted, then transferred to dry ice for 10 min, subjected to one additional freeze-thaw cycle, and centrifuged at 16,000 × *g* for 20 min at 4 °C. Supernatant was transferred to a fresh conical tube and 25 μL of a 50% slurry FLAG-M2 magnetic beads (Sigma-Aldrich) were added. The mixture was incubated for 2 hr at 4 °C with end-over-end rotation. Beads were pelleted by centrifugation at 100 × *g* for 1 min, magnetized, supernatant was removed and transferred with 1 mL of lysis buffer to a fresh centrifuge tube. Beads were washed three times with 1 mL rinsing buffer (20 mM Tris-HCl pH 8.0, 2 mM CaCl_2_). After the last wash the beads were pelleted at 500 × g for 1 min and all the liquid was removed. The beads were re-suspended in 20 mM Tris-HCl pH 8.0 with 500 ng trypsin (T6567, Sigma-Aldrich) and incubated with end over end rotation at 37 °C for 4hr. The supernatant was then removed and the beads were re-suspended in 20 mM Tris-HCl pH 8.0 with 500 ng trypsin and incubated overnight at 37 °C. Following the overnight trypsin digestion the supernatant was removed, combined, formic acid was added to the peptides to 2% final concentration and then the peptides were stored at −20 °C before analysis.

### BirA Affinity Purification

For the BirA pull downs, 4 × 150 cm^2^ dishes of Myc-BirA, Myc-BirA-T7-SOCS2 or Myc-BirA-T7-SOCS6 293 T-REx cells were grown to 80% confluence then treated with 75 µM vanadate, 1 µM MLN4924, 1 µg/mL Doxycycline and 2 mM Biotin for 24 hr. At the end of the 24 hr they were washed twice in 20 mL PBS, scraped into PBS, pooled and collected by centrifugation at 5,000 × g for 5 min at 4 °C. Cell pellets were snap frozen in dry ice and stored at −80 °C until lysis. Pellets were lysed in 2 mL of RIPA lysis buffer (50 mM Tris-HCl pH 7.5, 150 mM NaCl, 1 mM EDTA, 1 mM EGTA, 1% NP-40, 0.1% SDS, 10 mM NaF, 250 µM vanadate,1x cOmplete mini EDTA-free protease inhibitor cocktail (Sigma-Aldrich), 250 U Benzonase Nuclease (Santa Cruz Biotechnology)) at 4 °C for 1 hr with agitation, then sonicated (10 sec on and 3 sec off a total of three times at 35% power) to disrupt visible aggregates. The lysate was centrifuged at 21000 × g for 30 min. Clarified supernatants were incubated with 30 μL packed, pre-equilibrated streptavidin-Sepharose high performance beads (GE) with end over end agitation at 4 °C for 3 hr. Beads were collected by centrifugation 400× g 1 min, supernatant was removed and transferred with 1 mL of RIPA lysis buffer to a fresh centrifuge tube. Beads were washed one more time with RIPA, two times with 1 mL TAP buffer (50 mM HEPES-KOH pH 8.0, 100 mM KCl, 10% glycerol, 2 mM EDTA, 0.1% NP-40) and washed three times with 50 mM ammonium bicarbonate pH 8.0. After the last wash the beads were pelleted at 500 × g for 1 min and all the liquid was removed. The beads were re-suspended in 50 mM ammonium bicarbonate pH 8.0 with 1 µg trypsin (T6567, Sigma-Aldrich) and incubated at 37 °C with end over end agitation overnight. The next morning 500 ng of trypsin was added to the beads and incubated at 37 °C for two more hours. The supernatant was removed from the beads, the beads were washed two times with mass spec grade water. The washes and supernatant were combined and lyophilized in the speed vac. The lyophilized peptides were cleaned using ZipTip™ C18 (Millipore Corporation) before the MS analysis.

### Affinity Purification Mass Spectrometry

#### LC-MS/MS

LC-MS/MS analysis was performed with an Easy-nLC 1000 (Thermo Scientific) coupled to an Orbitrap Elite mass spectrometer (Thermo Scientific). The LC system configured in a vented format^102^ consisted of a fused-silica nanospray needle (PicoTip™ emitter, 75 µm ID, New Objective) packed in-house with Magic C18 AQ 100 Å reverse-phase media (Michrom Bioresources Inc.) (25 cm), and a trap (IntegraFrit™ Capillary, 100 µm ID, New Objective) containing Magic C18 AQ 200 Å (2 cm). The peptide sample was diluted in 10 µL of 2% acetonitrile and 0.1% formic acid in water and 8 µL was loaded onto the column and separated using a two-mobile-phase system consisting of 0.1% formic acid in water (A) and 0.1% acetic acid in acetonitrile (B). A 90 min gradient from 7% to 35% acetonitrile in 0.1% formic acid at a flow rate of 400 nL/minute was used for chromatographic separations. The mass spectrometer was operated in a data-dependent MS/MS mode over the m/z range of 400–1800. The mass resolution was set at 240,000. For each cycle, the 20 most abundant ions with +2 and +3 charges states from the scan were selected for MS/MS analysis using 35% normalized collision energy. Selected ions were dynamically excluded for 15 seconds.

#### Max Quant Data Search

The MaxQuant peptide identification and label-free quantification was performed as previously described with the following parameters^103^. The UniProt Reviewed 9606 human proteome and the contaminats.fasta were used for searching. Variable modifications: Oxidation (M), Acetyl (Protein N-term), and Biotinylated (K). Fixed Modifications Carbamidomethyl (C). Enzyme Trypsin with 2 maximum missed cleavages. Peptide false discovery rate 0.01. Minimum peptide length 7 and maximum peptide mass 4600. Minimum delta score for unmodified peptides 0 and minimum score for unmodified peptides 0. Minimum delta score for modified peptides 6 and minimum score for modified peptides 40.

#### Data Filtration

The average of the top three peptide intensities were used to estimate the protein abundance in each biological replicate. The abundance of each protein from each biological replicate was then run through the SAINTq software using the default settings^37^. Flag interacting proteins in Fig. 1 had a SAINT score >0.666, corresponding to a Bayesian false discovery rate <7.7%. BirA interacting proteins in Fig. 1 had a SAINT score >0.675, corresponding to a Bayesian false discovery rate <10%.

The top interactions were then imported into Cytoscape and the STRING database was used to determine the interactions between the interacting proteins.

## Electronic supplementary material

Supplementary Dataset

Supplementary material
