# Supplementary material for: SOCS2 Binds to and Regulates EphA2 through Multiple Mechanisms
Source: Sci Rep. 2017 Sep 7;7:10838. doi: 10.1038/s41598-017-11040-3 (PMC5589800; doi:10.1038/s41598-017-11040-3)

Supplementary material for

SOCS2 Binds to and Regulates EphA2 through Multiple Mechanisms

Carissa Pilling and Jonathan A. Cooper

Supplementary Figure 1:
Ezrin, EphA2, Crk, IRS4, β-Catenin, and CRL5 are strongly biotinylated by BirA-SOCS2

Supplementary Figure 2:
Myc-EphA2^{K646M} is not autophosphorylated in shControl or shEphA2 HeLa cells.

Supplementary Figure 3:
Single YF mutations in the EphA2 kinase domain do not disrupt binding between EphA2 and SOCS2

Supplementary Figure 4:
EphA2 pY588 antibody is specific but the pY771 antibody picks up background bands

Supplementary Figure 5:
EphA2 and EfnA1 expression levels in different cell lines, and stimulation of EphA2 phosphorylation by EfnA1-Fc at 0°C

Supplementary Figure 6:
EphA2 and EfnA1-Fc co-localize

Supplementary Figure 7:
Uncropped blots for Figure 2 panels a-f

Supplementary Figure 8:
Uncropped blots for Figure 3 panels b, d and f

Supplementary Figure 9:
Uncropped blots for Figure 4 panels a and c

Supplementary Figure 10:
Uncropped blots for Figure 7 panels a-d

Supplementary Figure 11:
Uncropped blots for Figure 7 panel a

Supplement Figure 1:

Ezrin, EphA2, Crk, IRS4, β-Catenin, and CRL5 are strongly biotinylated by BirA-SOCS2. HeLa cells were transiently transfected with either Myc-BirA, Myc-BirA-SOCS6^{WT}, Myc-BirA-SOCS2^{WT} or Myc-BirA-SOCS2^{R73K}. Twenty-four hours after transfection the cells were stimulated with 75 μM sodium orthovandate, 2 mM Biotin and 1 μM MLN4924 (where indicated) for twenty-four hours. Following the stimulation, the cells were lysed and biotinylated proteins were pulled out using streptavidin agarose beads. The whole cell extract represents 2% of the lysate used for the pull down.

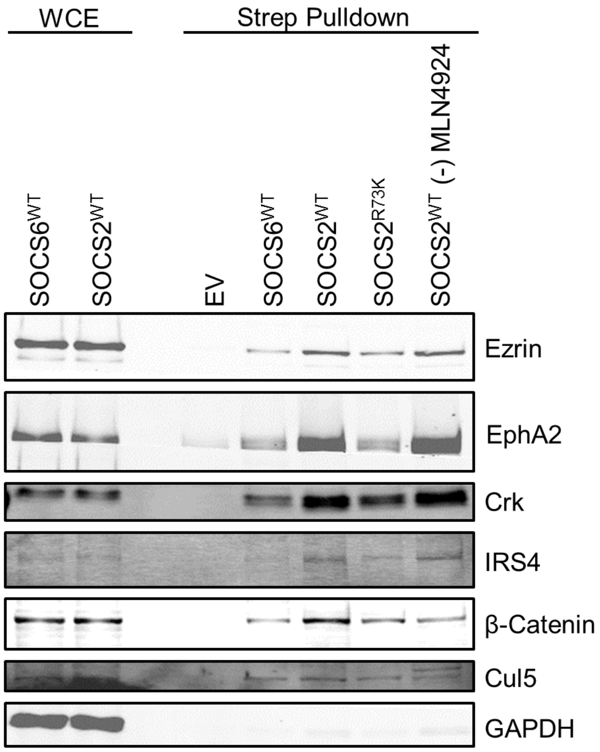

Supplement Figure 2:

Myc-EphA2^{K646M} is not autophosphorylated in shControl or shEphA2 HeLa cells.

HeLa shScrm or shEphA2 cells were transiently transfected with either Myc-EphA2^{WT} or Myc-EphA2^{K646M}. Twenty-four hours after transfection the cells were stimulated with 1 mM pervanadate for 30 min, lysed, immunoprecipitated with antibody to Myc and protein A/G beads. The whole cell extract represents 15% of the lysate used for the pull down.

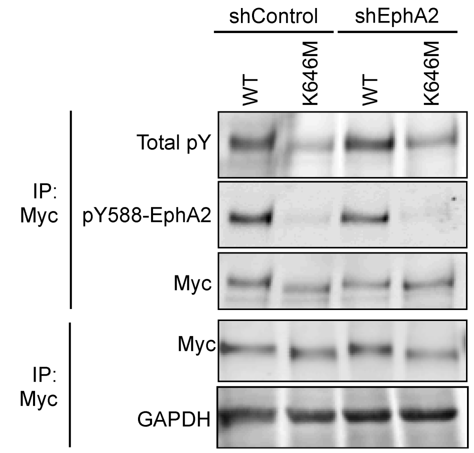

Supplement Figure 3:

Single YF mutations in the EphA2 kinase domain do not disrupt binding between EphA2 and SOCS2.

(a) Cartoon crystal structure of the EphA2 kinase domain (PDB: 1MQB), with every tyrosine displayed in green. (b, c) Eleven single tyrosine to phenylalanine point mutants in Myc-EphA2 can all bind to T7-SOCS2^{LCQQ}. HeLa cells were transiently transfected with T7-SOCS2^{LCQQ} and the indicated Myc-EphA2 construct. Twenty-four hours after transfection the cells were stimulated with 1 mM pervanadate for 30 min, lysed and immunoprecipitated with antibody to T7 and protein A/G beads. (d) The Myc-EphA2 Y729F, Y772F and Y729/772F mutants are all phosphorylated in 293T cells. 293T cells were transiently transfected with the indicated Myc-EphA2 construct. Twenty-four hours after transfection the cells were stimulated with 1 mM pervanadate for 30 min, lysed and immunoprecipitated with antibody to Myc and protein A/G beads. The whole cell extract represents the following percent of the lysate used for the pull down: panel a 10%, panel c 5% and panel d 7.5%.

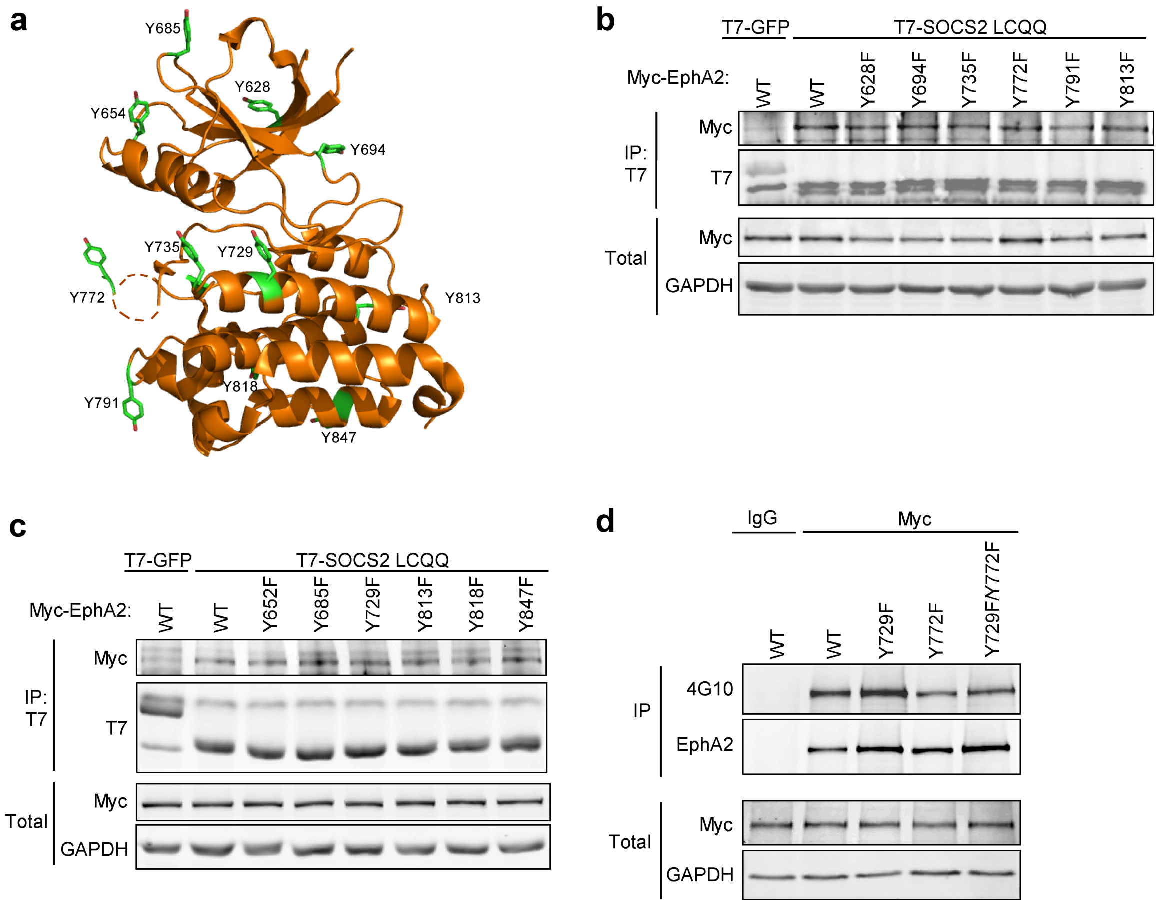

Supplement Figure 4:

EphA2 pY588 antibody is specific but the pY771 antibody picks up background bands.

(a) Analysis of the specificity of pY588 and pY772 EphA2 antibodies in whole cell extract. HeLa shScrm or shEphA2 cells were starved in DMEM 0.5% BSA 10 mM HEPES for 16 hrs then stimulated with 1 µg/mL EprinA1-Fc or left un-stimulated for 5 min. The cells were lysed and analyzed with the indicated antibodies. (b) The pY588 and pY772-EphA2 antibodies are specific for their site when EphA2 is immunoprecipitated. HeLa cells were transiently transfected with the indicated Myc-EphA2 constructs. Twenty-four hours after transfection the cells were stimulated with 1 mM pervanadate for 30 min, lysed and immunoprecipitated with antibody to Myc and protein A/G beads.

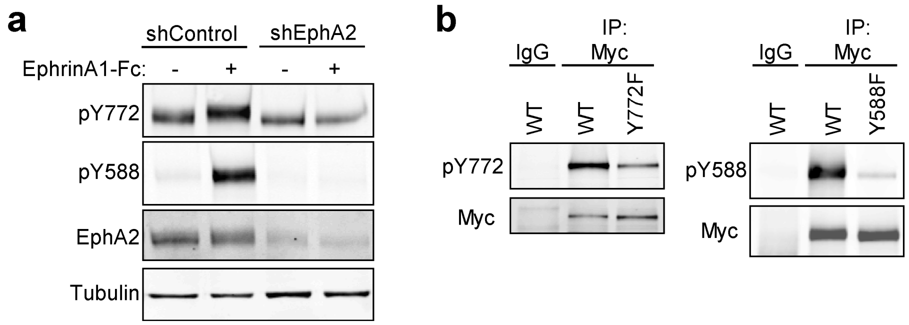

Supplement Figure 5:

EphA2 and EfnA1 expression levels in different cell lines, and stimulation of EphA2 phosphorylation by EfnA1-Fc at 0°C

(a) MDA-MD-231 cells have high EphA2 and low EfnA1 RNA compared to MCF10A and HeLa cells. RNA was harvested from un-stimulated HeLa, MCF10A and MDA-MB-231 cells. A SYBR green qPCR was performed on the cDNA from each of these cells lines and gene expression was calculated using the ΔΔCt method. Bars are mean and standard deviation of three biological independent experiments. (b) EfnA1 stimulates EphA2 phosphorylation at 0°C. MDA-MB-231cells were starved in DMEM, 0.5% BSA, 10 mM HEPES for 4 hr followed by a 1 hr incubation with EfnA1-Fc or Fc (1 μg/mL) on ice. The ligand was washed off and the cells were placed at 37°C for the indicated times. Cells were lysed and immunoprecipitated with antibody to EphA2 and protein A/G beads.

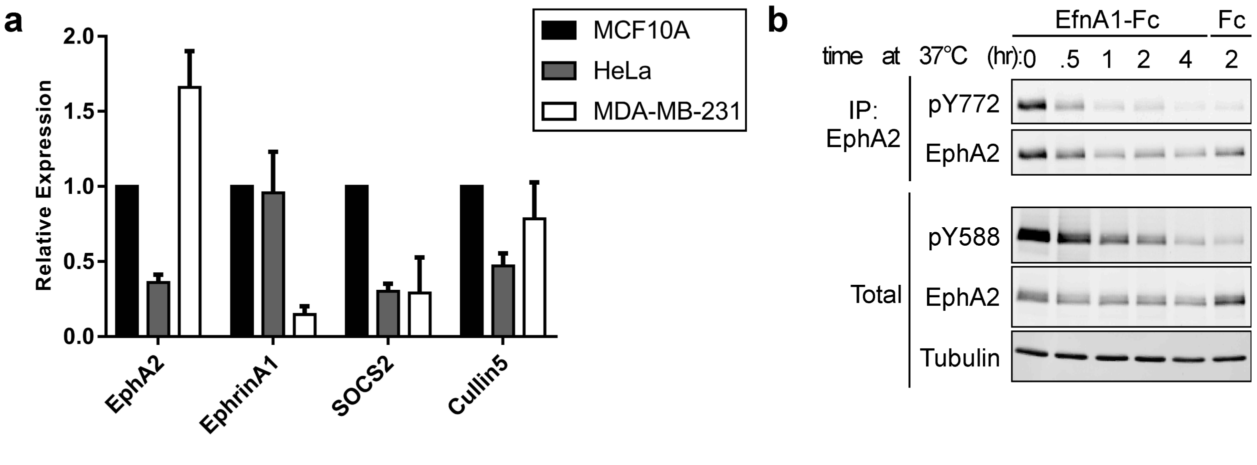

Supplement Figure 6:

EphA2 and EfnA1-Fc co-localize.

MDA-MB-231 cells were starved in DMEM 0.5% BSA 10 mM HEPES for 4 hrs followed by a 1 hr incubation with EfnA1-Fc (1 μg/mL) on ice. The ligand was washed off the cells and the cells were moved to 37°C for the indicated amount of time before fixation. Fixed and permeabilized cells were stained with anti-EphA2, anti-Fc and appropriate secondary antibodies. Images are maximum intensity projections of three Z-sections. The EphA2 and EfnA1 brightness/contrast is unequal in some images to allow for easy visualization. Scale bar: 15 μm.

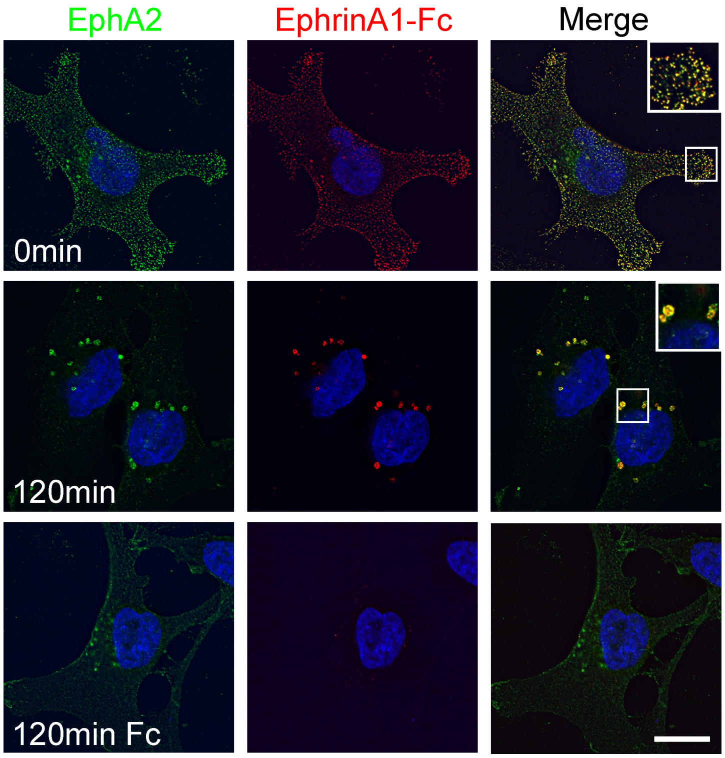

Supplement Figure 7:

Uncropped blots for Figure 2 panels a-f.

Membranes were cut at the positions indicated before probing with various antibodies.

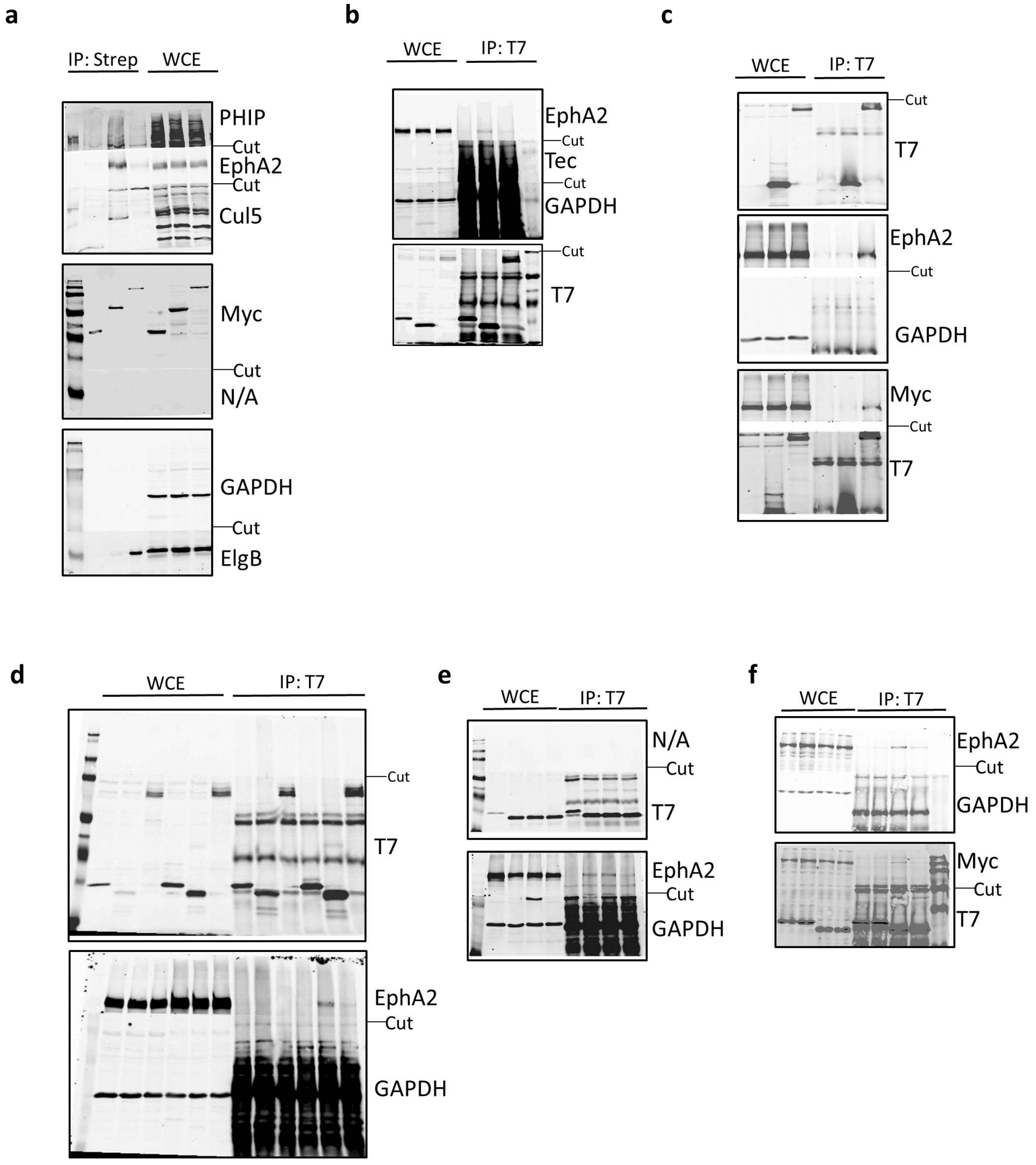

Supplement Figure 8:

Uncropped blots for Figure 3 panels b, d and f.

Membranes were cut at the positions indicated before probing with various antibodies.

b

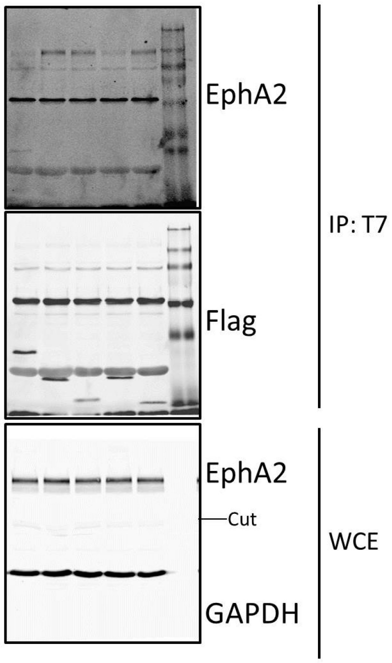

d

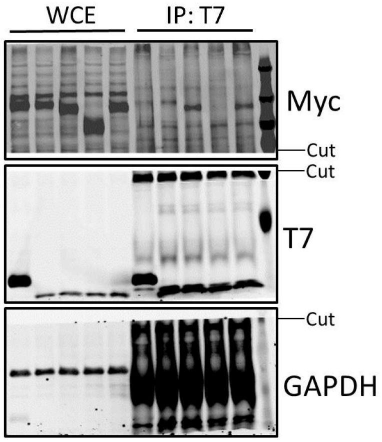

f

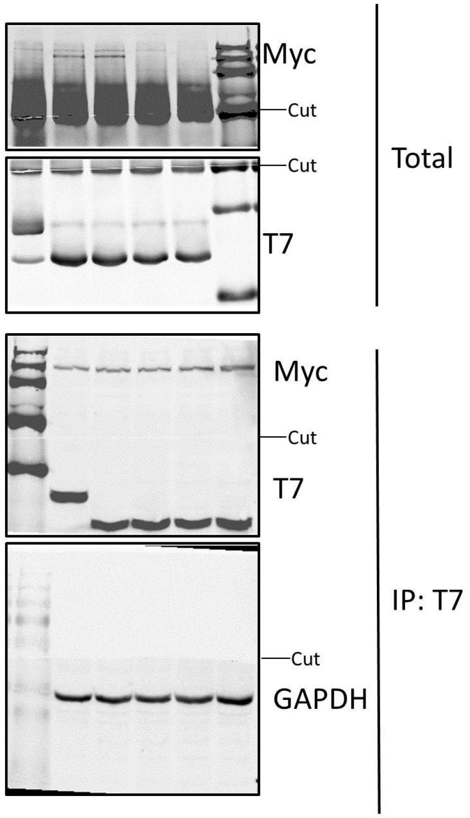

Supplement Figure 9:

Uncropped blots for Figure 4 panels a and c.

Membranes were cut at the positions indicated before probing with various antibodies.

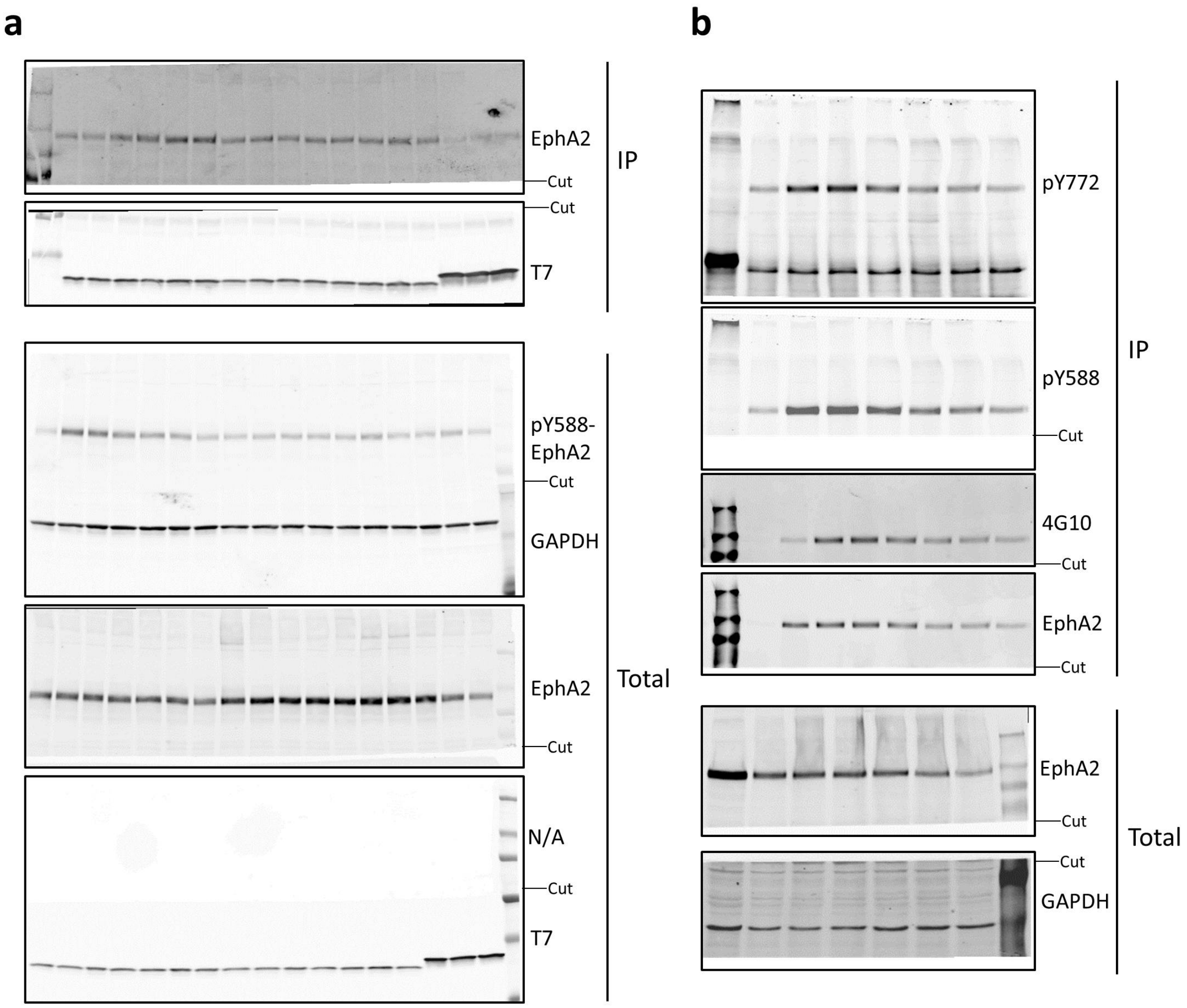

Supplement Figure 10:

Uncropped blots for Figure 7 panels a-d.

Membranes were cut at the positions indicated before probing with various antibodies.

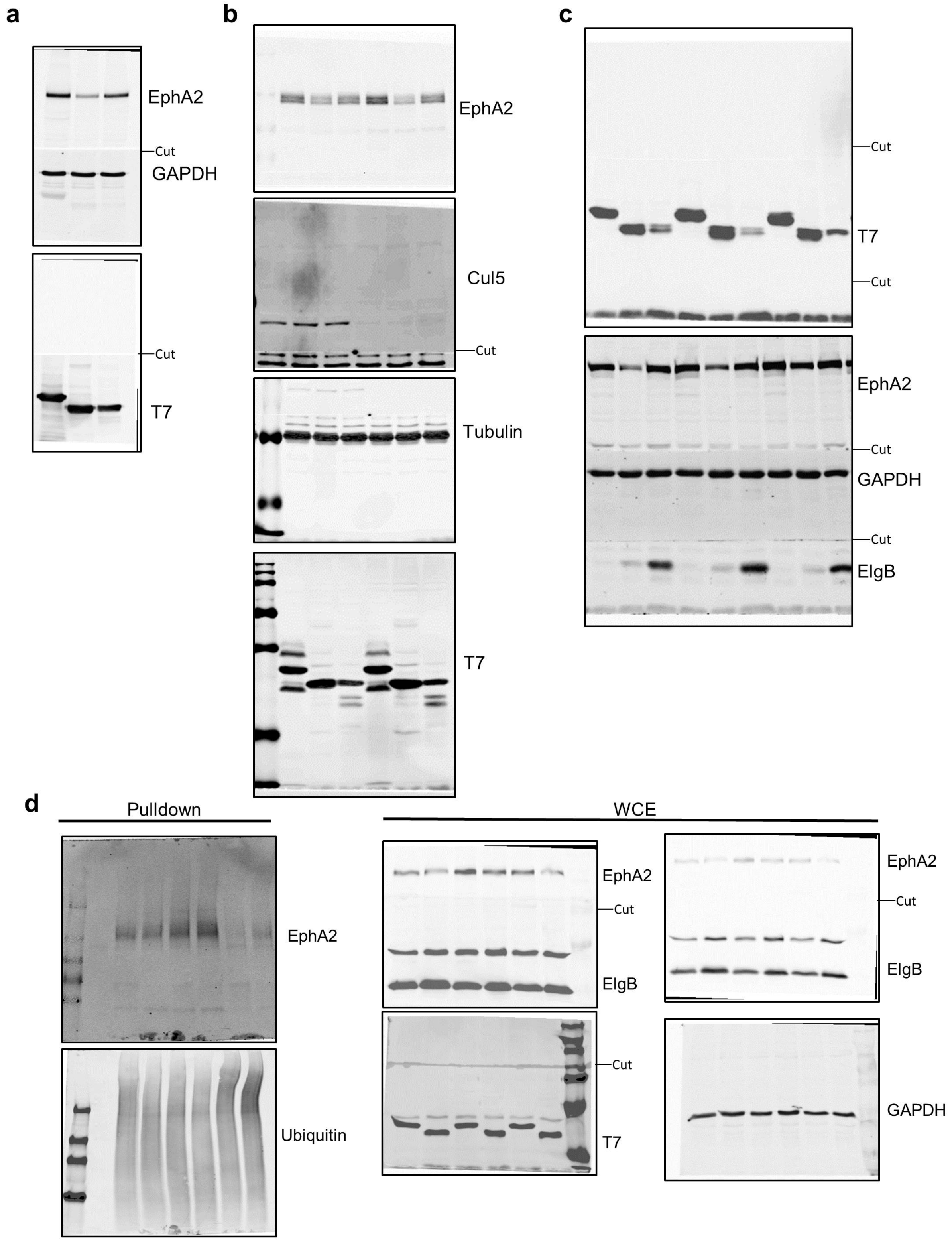

Supplement Figure 11:

Uncropped blots for Figure 7 panel a.

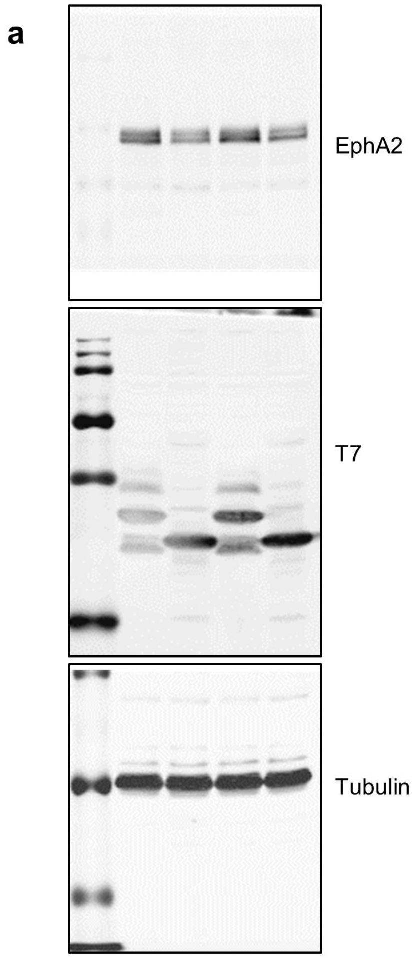

Supplement: Supplementary file 2 — Supplementary material [file 41598_2017_11040_MOESM2_ESM.pdf]
